# Resistance of *Dickeya solani* strain IPO 2222 to lytic bacteriophage ΦD5 results in fitness tradeoffs for the bacterium during infection

**DOI:** 10.1038/s41598-022-14956-7

**Published:** 2022-06-24

**Authors:** Przemyslaw Bartnik, Kinga Lewtak, Marta Fiołka, Paulina Czaplewska, Magdalena Narajczyk, Robert Czajkowski

**Affiliations:** 1 Laboratory of Biologically Active Compounds, Intercollegiate Faculty of Biotechnology UG and MUG, University of Gdansk, Antoniego Abrahama 58, 80-307 Gdansk, Poland; 2 Department of Cell Biology, Institute of Biological Sciences, Maria Curie-Sklodowska University, Akademicka 19, 20-033 Lublin, Poland; 3 Department of Immunobiology, Institute of Biological Sciences, Maria Curie-Sklodowska University, Akademicka 19, 20-033 Lublin, Poland; 4 Laboratory of Mass Spectrometry‐Core Facility Laboratories, Intercollegiate Faculty of Biotechnology UG and MUG, University of Gdansk, Antoniego Abrahama 58, Gdansk, Poland; 5 Laboratory of Electron Microscopy, Faculty of Biology, University of Gdansk, Wita Stwosza 59, 80‐308 Gdansk, Poland

**Keywords:** Molecular biology, Microbiology, Bacteriology, Bacteriophages, Environmental microbiology

## Abstract

Resistance to bacteriophage infections protects bacteria in phage-replete environments, enabling them to survive and multiply in the presence of their viral predators. However, such resistance may confer costs for strains, reducing their ecological fitness as expressed as competitiveness for resources or virulence or both. There is limited knowledge about such costs paid by phage-resistant plant pathogenic bacteria in their natural habitats. This study analyzed the costs of phage resistance paid by the phytopathogenic pectinolytic bacterium *Dickeya solani* both in vitro and in potato (*Solanum tuberosum* L.) plants. Thirteen Tn5 mutants of *D. solani* IPO 2222 were identified that exhibited resistance to infection by lytic bacteriophage vB_Dsol_D5 (ΦD5). The genes disrupted in these mutants encoded proteins involved in the synthesis of bacterial envelope components (viz. LPS, EPS and capsule). Although phage resistance did not affect most of the phenotypes of ΦD5-resistant *D. solani* such as growth rate, production of effectors, swimming and swarming motility, use of various carbon and nitrogen sources and biofilm formation evaluated in vitro, all phage resistant mutants were significantly compromised in their ability to survive on leaf surfaces as well as to grow within and cause disease symptoms in potato plants.

## Introduction

Bacteriophages (bacterial viruses) are the most abundant biological entities in the biosphere. With their total estimated number of ca. 10^31^ phage particles, they are the main driving force of bacterial adaptation and evolution^1–4^. Likewise, bacterial viruses play a critical role in maintaining bacterial diversity in the environment^5–7^. It is estimated that up to 20% of bacterial cells are killed daily due to phage infections among all habitats^8^. However, surprisingly, this enormous mortality of bacterial cells does not lead to the global disappearance of sensitive host populations or a dominance of resistant ones in a given ecological niche^9–11^. On the contrary, phage-susceptible and phage-resistant bacterial populations are frequently reported to coexist both in natural (e.g. ocean, phyllosphere)^12,13^ as well as human-controlled and engineered environments (e.g. wastewater treatment facilities, agricultural fields)^14–16^.

It is hypothesized that the stable coexistence of phage-resistant and phage-susceptible bacterial populations occurs because bacteria pay a cost for phage resistance, irrespective of whether the viruses are present in the environment or not^17–20^. The altered fitness of resistant bacteria is usually manifested by reduced competitiveness for resources and/or reduced virulence or both^21,22^. However, it should also be highlighted that not all strains that have evolved phage resistance suffer such costs^23,24^. Such costs may also depend on the environmental context of the bacterium as well as the mechanisms by which phage resistance is conferred^25,26^.

Even though the costs of resistance to phage infection have been reported in various phage-host systems^20,27,28^, relatively little is known about these costs paid specifically by phage-resistant phytopathogenic bacteria residing in their natural habitats, in agricultural fields. This is an important issue given the diversity of conditions such bacteria encounter in these settings^29^. Indeed, no such studies have addressed the costs of phage resistance in Soft Rot *Pectobacteriaceae* (SRP) species which, due to their life-style, have particularly complex interactions with varied habitats.

SRP bacteria including *Pectobacterium* spp. and *Dickeya* spp. are an excellent model for studying phage-host interaction and co-adaptation in the environment. These bacteria are considered among the ten most important agricultural phytopathogens worldwide^30^. SRP cause significant losses in crop production (up to 40%), with disease severity dependent on weather conditions, plant susceptibility, pathogen inoculum, and other factors^29,31^. *Pectobacterium* spp. and *Dickeya* spp. are widespread in various ecological niches, including natural and agricultural soils, water, sewage, the surface of host and non-host plants, and the surface and interior of insects^32–34^. Because of the diverse environments in which SRP bacteria are found, these pathogens apparently experience ecological and life-style tradeoffs because of their frequent transfer between these diverse environments: for example, from host plant to soil, plant to plant, host plant to non-host plant, surface/irrigation water to plant and vice versa^29^. In all of these settings, SRP bacteria can encounter lytic bacteriophages and, as a result, may become repeatedly infected^35^. Under conditions of high infection, resulting in large populations of the pathogen, the emergence of phage and subsequently, phage-resistant SRP variants, is inevitable^36,37^. However, it remains unclear what the ecological fitness costs are for SRP bacteria to become resistant to such viral infections and thus whether phage infection can reduce disease severity both directly by, at least temporarily, reducing pathogen populations or subsequently by reducing pathogen virulence or fitness.

The purpose of this study was to assess the fitness costs paid by phage-resistant variants of *D. solani* both in vitro and *in planta*. *D. solani* is an emerging plant pathogen that causes soft rot disease symptoms in a variety of crops and nonfood plants worldwide^38,39^. This species was first reported to infect potato in the early 2000s^40^ and has since become a serious worldwide problem in agriculture. At present, *D. solani* is an important pathogen in a majority of European countries^38,39^ as well as in Israel^41^, Georgia^42^, Turkey^43^ and Brazil^44^ causing large losses in crop production. Surprisingly, most of the *D. solani* strains analyzed so far belong to the same haplophyte and express only a small number of genetic differences^45^.

Our study investigated the interaction of *D. solani* strain IPO 2222^38^, the type strain of this species, that has received considerable attention, with bacteriophage vB_Dsol_D5 (ΦD5)^46,47^. ΦD5 is a broad host range lytic bacteriophage able to infect *D. solani* strains as well as those of 3 other *Dickeya* species (*D. zeae*, *D. dianthicola* and *D. dadantii*)^46^. Phage ΦD5 is in the genus *Limestonevirus* and family *Ackermannviridae*^48–50^. Of all bacteriophages infecting *D. solani*, those within the *Limestonevirus* genus are the most abundant in the environment^50^. Limestoneviruses, including ΦD5, have been isolated in various European countries, including Belgium, Poland, Russia and the United Kingdom and exhibit a high level of genetic homogeneity^50^. Furthermore, ΦD5 has been extensively studied as a biological control agent useful in the control of disease caused by *D. solani* in the potato ecosystem^47^.

Using random Tn5-based mutagenesis we identified *D. solani* genes and operons that encoded structures required for ΦD5 attachment and susceptibility to infection to better understand the molecular determinants of phage-resistant bacterial variants as well as the fitness of mutants. We thus have addressed the hypothesis that the level of fitness tradeoff conferred by phage resistance is dependent on the environmental context in which fitness is assessed.

## Results

### Identification of disrupted genes in ΦD5-resistant D. solani mutants

**Figure 1:**
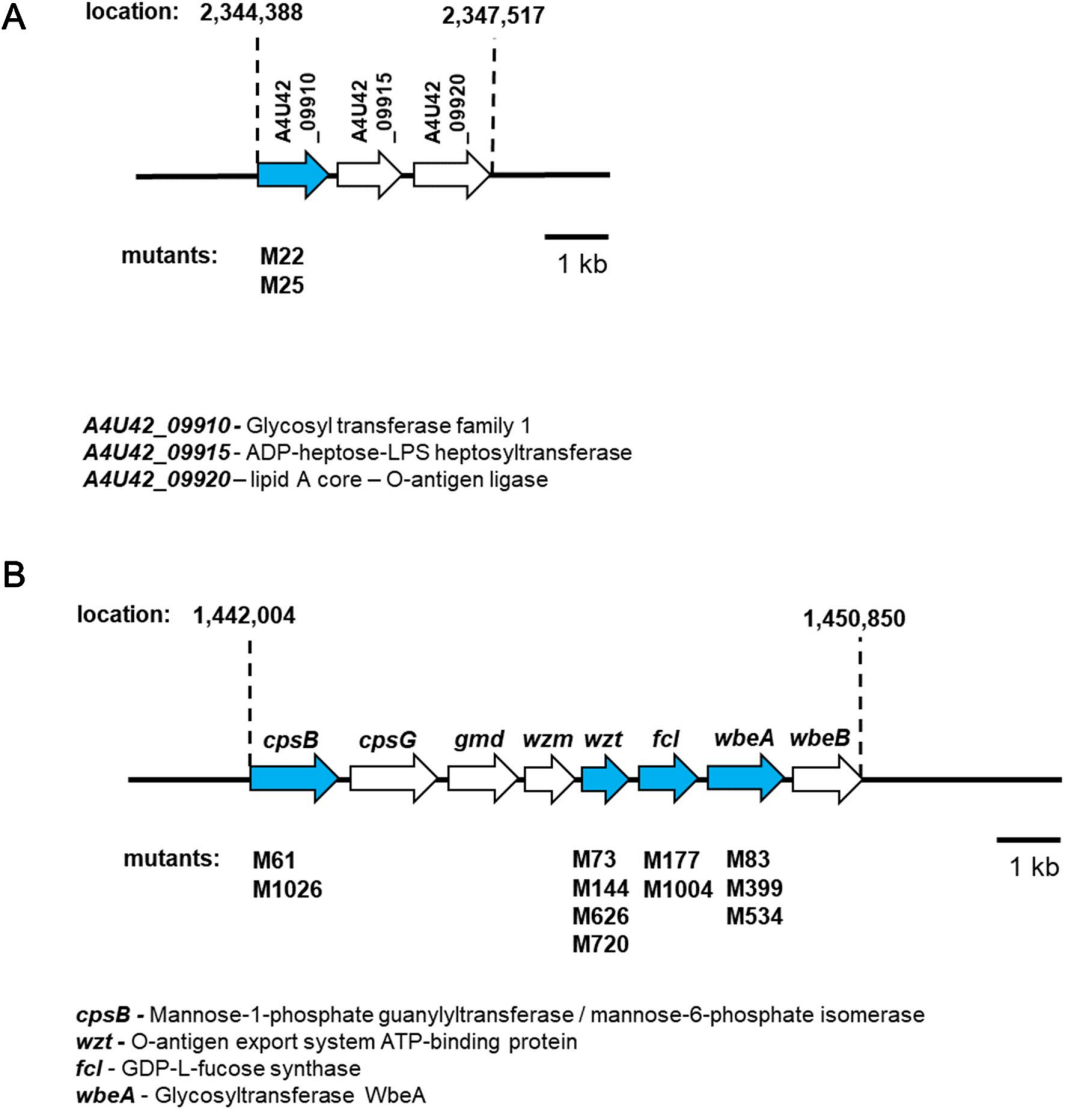
Two operons of *D. solani* involved in the interaction of the bacterium with lytic bacteriophage ΦD5. (**A**) Operon associated with *rfa* gene cluster involved in the biosynthesis of the core region of LPS in Gram-negative bacteria and (**B**) Putative O-antigen LPS biosynthesis cluster^51^. The *D. solani* ORFs affected by the Tn5 insertion are marked in blue. The directions of the arrows represent the direction of the transcription.

A total of 1000 Tn5 mutants of *D. solani* was screened for resistance to infection by bacteriophage ΦD5. Thirteen mutants (ca. 1.3%) were found to be resistant to this phage (Table 1). The genomes of these mutants were sequenced to identify the Tn5 insertion sites. A single insertion of Tn5 was found in each of the ΦD5-resistant mutants analyzed. Interestingly, in multiple cases distinct Tn5 insertions were found in the same locus (Fig. 1, Table 1); a gene encoding glycosyltransferase family 1 was disrupted in both mutants M22 and M25; mannose-1-phosphate guanylyl-transferase/ mannose-6-phosphate isomerase was disrupted in mutants M61 and M1026; a sugar ABC transporter ATP-binding protein was disrupted in mutants M73, M144, M626 and M720; a hypothetical protein homologous to protein WbeA involved in O-antigen transport system was disrupted in mutants M83, M399 and M534 and a GDP-fucose synthase was disrupted in mutants M177 and M1004 (Table 1, Fig. 1). In total, five distinct bacterial loci were required for infection of *D. solani* with ΦD5. All loci disrupted by Tn5 encoded proteins associated with synthesis, metabolism, storage and/or modification of bacterial surface features, including LPS, EPS and capsular polysaccharide (Table 1, Fig. 1). The sequences (ca. 1000–5000 bp.) bordering these five loci were analyzed using BlastP to obtain additional insights into their genomic context and transcriptional organization. All five loci were associated with a total of only 2 operons (Fig. 1, Table 1). One operon is a component of the *rfa* gene cluster involved in the biosynthesis of the core region of LPS (mutants M22 and M25). The second operon encodes an O-antigen LPS biosynthesis cluster^51^ (mutants M61, M73, M83, M144, M177, M399, M534, M626, M720, M1004 and M1026). Examination of the KEGG pathways corresponding to these 5 transcriptional units enabled their assignment to the cellular pathways involved in the biosynthesis of cell surface lipopolysaccharides and exopolysaccharides. Likewise, the putative interacting partners of the these 5 proteins assessed using STRING revealed that the products of all 5 loci interact with proteins associated with bacterial cell surface features (involved in the synthesis and remodeling of the capsule, LPS and EPS) (Supplementary Table 2). These 5 loci are all conserved in other *Dickeya* species, with homologs found in *D. dianthicola*, *D. dadantii*, *D. fangzhongdai*, *D. zeae*, *D. oryzae*, *D. unidicola* and *D. chrysanthemi* strains (Supplementary Table 2).

**Table 1:** Genetic loci of *Dickeya solani* strain IPO 2222 Tn5 mutants expressing resistance against phage vB_Dsol_D5 (ΦD5) .

No	Mutant	Insertion name, Tn5 locus, gene, CDC	Protein name	Gene length (nt)/protein length (aa)	Genomic context of the Tn5 insertions (Tn5 mutated gene marked in bold) ^A^	Entry, KEGG pathway, UniProt-based protein function
1	M22, M25	*p22*, *p25,* A4U42_09910, ANE75629.1	glycosyl transferase family 1	1125 nt/374 aa	Operon (3 genes): lipid A core—O-antigen ligase ADP-heptose: LPS heptosyltransferase glycosyltransferase family 1	EC: entry not assigned, gene not included in the pathway, O-antigen synthesis and metabolism, a protein involved in cell wall biogenesis
2	M61, M1026	*p61, p1026,* *cpsB*, (*manC*) A4U42_06115, ANE74940.1	mannose-1-phosphate guanylyltransferase/mannose-6-phosphate isomerase	1398 nt/465 aa	Operon (8 genes): glycosyl transferase (*wbeB*) hypothetical protein (putative *wbeA*) GDP-D-mannose dehydrogenase (*gmd*) phosphomannomutase (*cpsG*) mannose-1-phosphate guanylyltransferase (*cpsB*) O-antigen export system ATP-binding protein (*wzt*) O-antigen export system permease protein (*wzm*) nucleoside-diphosphate-sugar epimerase	EC: 2.7.7.13, O‐antigen nucleotide sugar biosynthesis, amino sugar, and nucleotide sugar metabolism, capsular polysaccharide colanic acid biosynthesis protein; catalyzes the formation of GDP-mannose from GTP and alpha-D-mannose 1-phosphate; colanic acid biosynthesis pathway
3	M73, M144, M626, M720	*p73, p144, p626, p720* *wzt*, (*rbfB*) A4U42_06135, ANE74944.1	O-antigen export system (LPS transport system) ATP-binding protein	732 nt/243 aa	Operon (8 genes): glycosyltransferase (*wbeB*) hypothetical protein (putative *wbeA*) GDP-D-mannose dehydrogenase (*gmd*) phosphomannomutase (*cpsG*) mannose-1-phosphate guanylyltransferase (*cpsB*) O-antigen export system ATP-binding protein (*wzt*) O-antigen export system permease protein (*wzm*) nucleoside-diphosphate-sugar epimerase	EC: entry not assigned, gene not included in the pathway, ABC transporter for LPS, nitrate ABC transporter, ATP-binding protein;
4	M83, M399, M534	*p83, p399, p534,* *wbeA*, A4U42_06145, ANE74946.1	hypothetical protein (putative glycosyltransferase WbeA)	1200 nt/399 aa	Operon (8 genes): glycosyl transferase (*wbeB*) hypothetical protein (putative *wbeA*) GDP-D-mannose dehydrogenase (*gmd*) phosphomannomutase (*cpsG*) mannose-1-phosphate guanylyltransferase (*cpsB*) O-antigen export system ATP-binding protein (*wzt*) O-antigen export system permease protein (*wzm*) nucleoside-diphosphate-sugar epimerase	EC: entry not assigned, gene not included in the pathway, LPS synthesis and maturation
5	M177, M1004	*p177, p1004,* *fcl*, A4U42_06140, ANE74945.1	GDP-L-fucose synthase	939 nt/312 aa	Operon (8 genes): glycosyl transferase (*wbeB*) hypothetical protein (putative *wbeA*) GDP-D-mannose dehydrogenase (*gmd*) phosphomannomutase (*cpsG*) mannose-1-phosphate guanylyltransferase (*cpsB*) O-antigen export system ATP-binding protein (*wzt*) O-antigen export system permease protein (*wzm*) GDP-L-fucose synthase (*fcl*)	EC: 1.1.1.271, O‐antigen nucleotide sugar biosynthesis, amino sugar, and nucleotide sugar metabolism, epimerase domain-containing protein

^**A**^Assessment of the transcriptional organization was predicted using Operon‐mapper (https://biocomputo.ibt.unam.mx/ operon_mapper/, assessed on 25 January 2022). The complete genome sequence of *D. solani* IPO 2222 WT (Genbank accession: CP015137.1^45^) was used as a reference.

### Adsorption of ΦD5 to WT and phage resistant mutants in vitro

**Figure 2:**
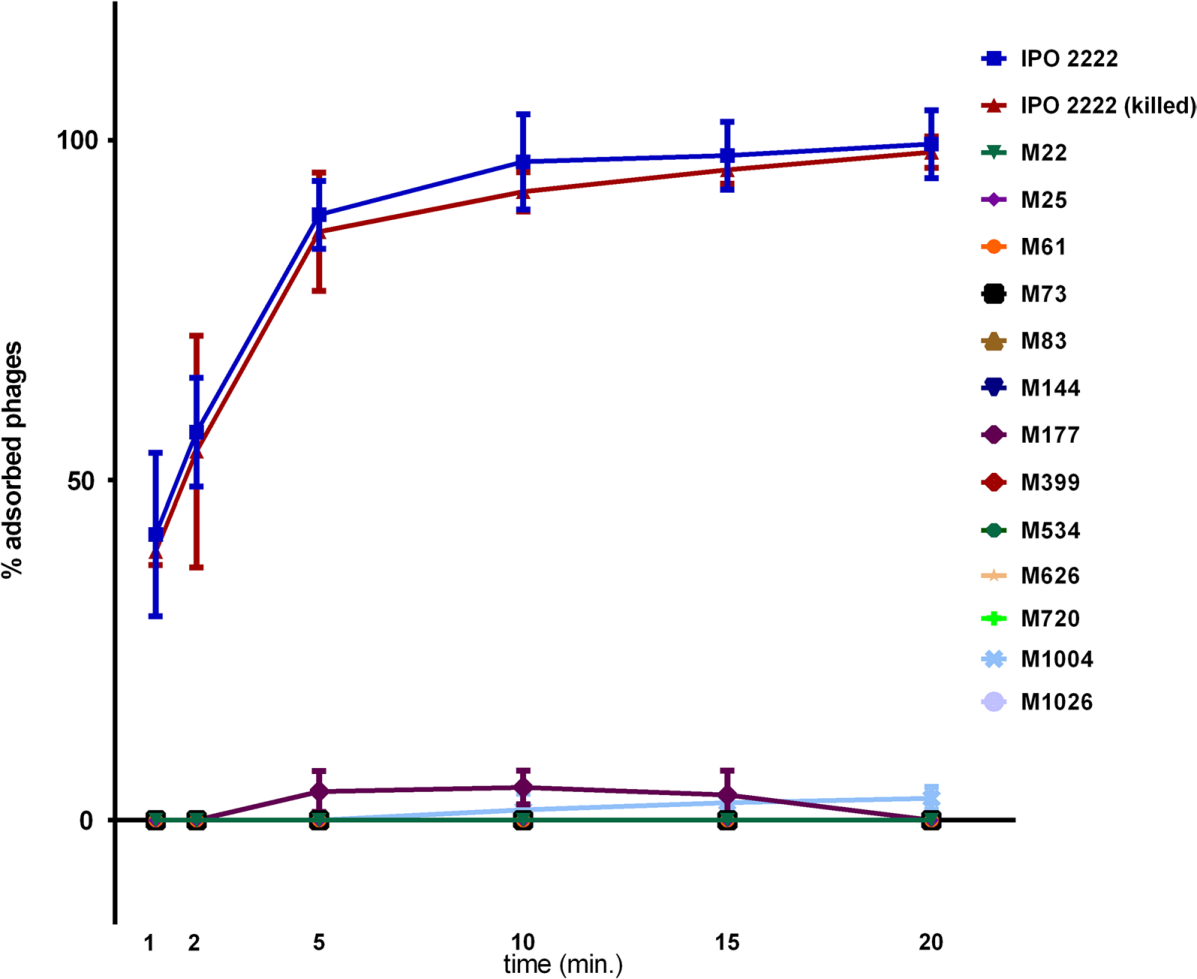
Adsorption of ΦD5 to viable and chloramphenicol-killed cells of WT and phage-resistant *D. solani* . A MOI of 0.01 of ΦD5 was used for adsorption assay and the total assay time was 20 min. Phage adsorption was calculated as follows: the percentage adsorption = (the average titer of unabsorbed phages per sample/average titer of phages in negative control) × 100. The averages and standard deviations of three independent repetitions per strain (WT or mutants) are shown.

**Figure 3:**
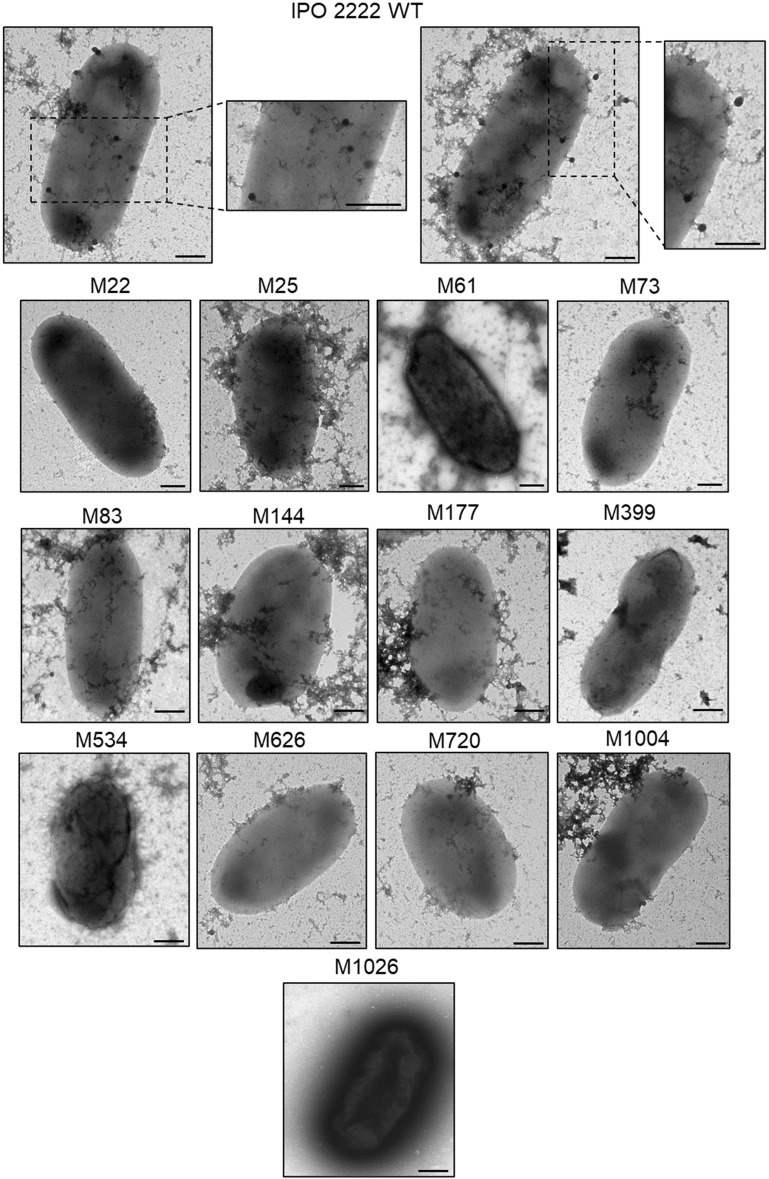
Visualization of adsorption of ΦD5 particles to WT and phage-resistant *D. solani* mutants by transmission electron microscopy (TEM). Bacterial cells and phage particles were mixed at MOI of 10 and incubated for 20 min at room temperature (ca. 20–22 °C) to allow the phages to attach to host bacterial cells. At least 10 individual images were gathered for each analyzed strain, and the experiment was repeated once (two biological replicates of the assay). Representative photos are shown. Scale bar—200 nm.

The adsorption of ΦD5 to both viable and dead (chloramphenicol-killed) cells of wild-type *D. solani* cells was rapid (Fig. 2). Within 5 min, nearly 90% of the phage particles had adsorbed to both living and dead cells of the WT strain and more than 99% had bound by 20 min. In contrast, the adsorption of ΦD5 to 11 phage-resistant mutants (M22, M25, M61, M73, M83, M144, M399, M534, M626, M720, M1026) was completely abolished, and only between 1 and 7% had bound to mutants M177 and M1004 within 20 min (Fig. 2). The lack of binding of ΦD5 to most mutants was confirmed using transmission electron microscopy (TEM); while abundant adsorption of the phage WT cells was observed, no adsorption of ΦD5 to phage-resistant Tn5 mutants occurred (Fig. 3).

### Biochemical phenotypes of phage-resistant mutants

**Figure 4:**
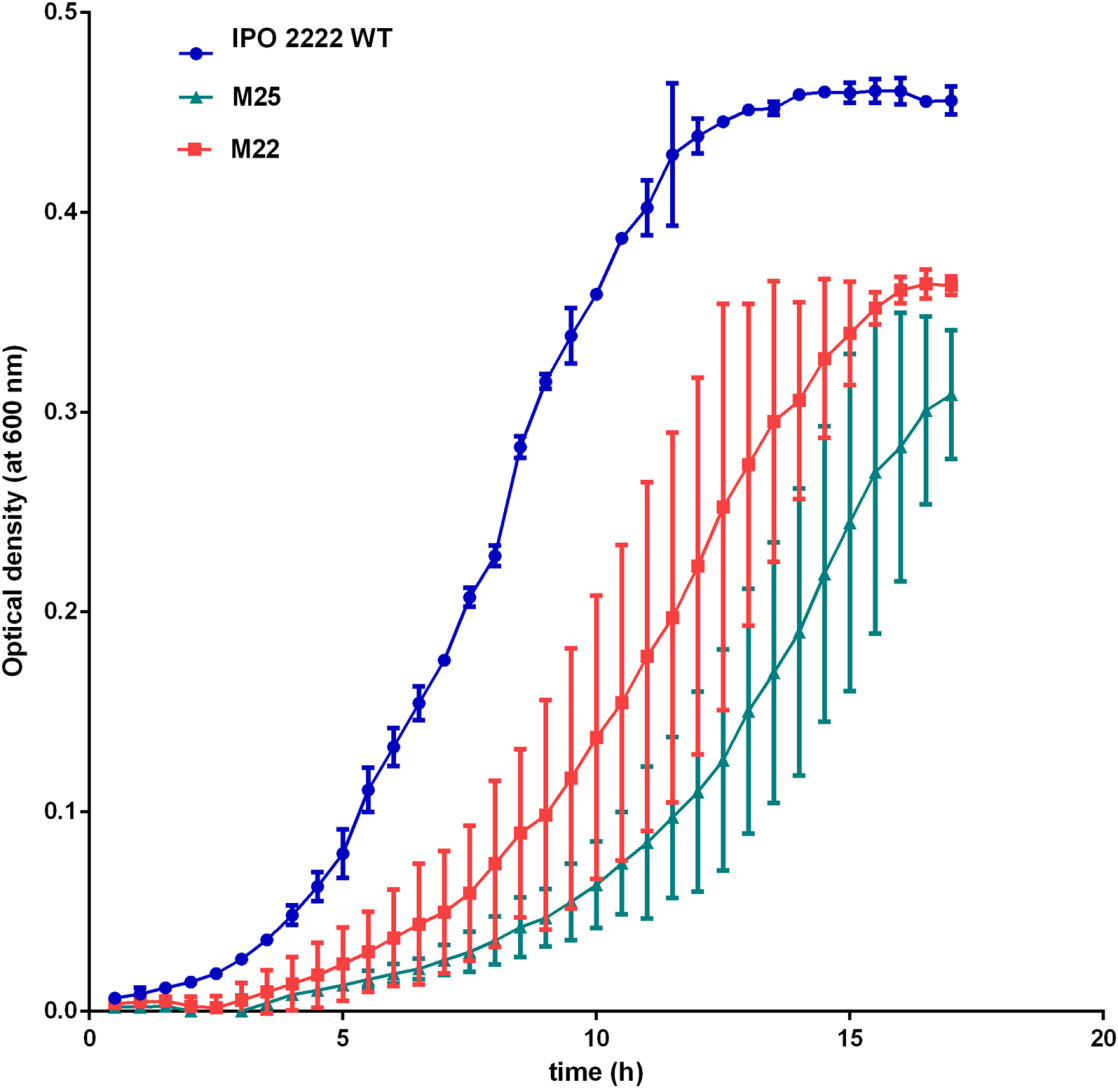
Growth of *D. solani* WT and phage-resistant mutants M22 and M25 in minimal growth medium (M9 + 0.4% glucose). The experiment was performed in two biological replicates containing two technical replicates each (n = 4). The results were averaged for presentation. The bars show standard deviation (SD).

The phage-resistant mutants were tested for changes in phenotypes that may be important for the ecological fitness and virulence of *D. solani* under natural and agricultural settings. No differences were found between the mutants and the WT strain in most metabolic phenotypes examined using BIOLOG GEN III and Ecoplate phenotypic microarrays. The ΦD5-resistant mutants differed from the WT strain in a total of only 3 features out of 125 tested using these systems. Mutants M73, M144, M626 and M720, having a disrupted ATP-binding protein in the O-antigen export system, lost the ability to utilize D-cellobiose as a sole carbon source, and mutants M22 and M25, having a disrupted glycosyltransferase family protein, became susceptible to troleandomycin. In addition, mutants M73, M144, M626 and M720 with a disrupted *wzt,* M83, M399 and M534 with a disrupted *wbeA,* and M177 and M1004 with a disrupted *fcl*, gained the ability to utilize inosine, a feature absent both in the WT and 4 other phage-resistant mutants (M22, M25, M61 and M1026) tested. All mutants shared with the WT strain the ability to produce cavities on CVP, produced proteases and degraded carboxymethylcellulose and polygalacturonic acid, while lacking the ability to produce siderophores or grow on TSA medium supplemented with 5% NaCl. Furthermore, while all mutants retained the ability to form some biofilm, all mutants except M22 and M25 produced more abundant biofilm than the WT strain. The LPS purified from the phage-resistant mutants was not distinguishable from that of the WT strain (Supplementary Fig. 1). No differences in cell morphology or size diameter were noted in the mutants compared to the WT strain by examination using transmission electron microscopy (Fig. 3). Likewise, all mutants exhibited similar colony morphology and colony diameter to that of the WT strain. None of the mutants differed significantly in their average generation times in rich (TSB) medium although M22 and M25 grew more slowly in a minimal medium (M9 + 0.4% glucose) compared to that of the WT strain (Fig. 4). The growth rate of the mutants over a range of temperatures (8, 15, 28, 37 °C) did not differ from that of the WT strain and none of the strains grew at either 4 or 42 °C. The growth rate of the mutants at pH 5.0 and pH 10.0 was similar to that of the WT strain (data not shown). While the WT was capable of both swimming and swarming motility, mutants M83, M177, M399, M534 and M1004 were nonmotile for both phenotypes and the other mutants exhibited reduced swimming motility compared to the WT strain. None of the mutants were capable of swarming motility. All mutants expressed similar susceptibility/resistance to all of the antibiotics tested as the WT strain, except for their resistance to kanamycin conferred by Tn5. All phage-resistant *D. solani* mutants were also significantly affected in their production of extracellular polymeric substances (EPS); M22, M25, M83, M399 and M534 produced less EPS (ca. 8 to 23% as much) than the WT strain. In contrast, mutants M61, M73, M144, M177, M626, M720, 1004 and M1026 produced more EPS (ca. 175–210% as much) than the WT strain.

### Differential permeability of outer membrane of phage mutants

Given that all ΦD5-resistant mutants had putative alterations in their envelope, their permeability was compared with that of the WT strain. The rate of death was much higher for the phage resistant mutants than the WT strain. While 70% of the cells of the WT strain survived exposure to a SDS solution for 1 min and ca. 15% survived for 10 min, only 30 to 60% of the mutants survived for even 1 min.

### Surface features of phage-resistant mutants

**Figure 5:**
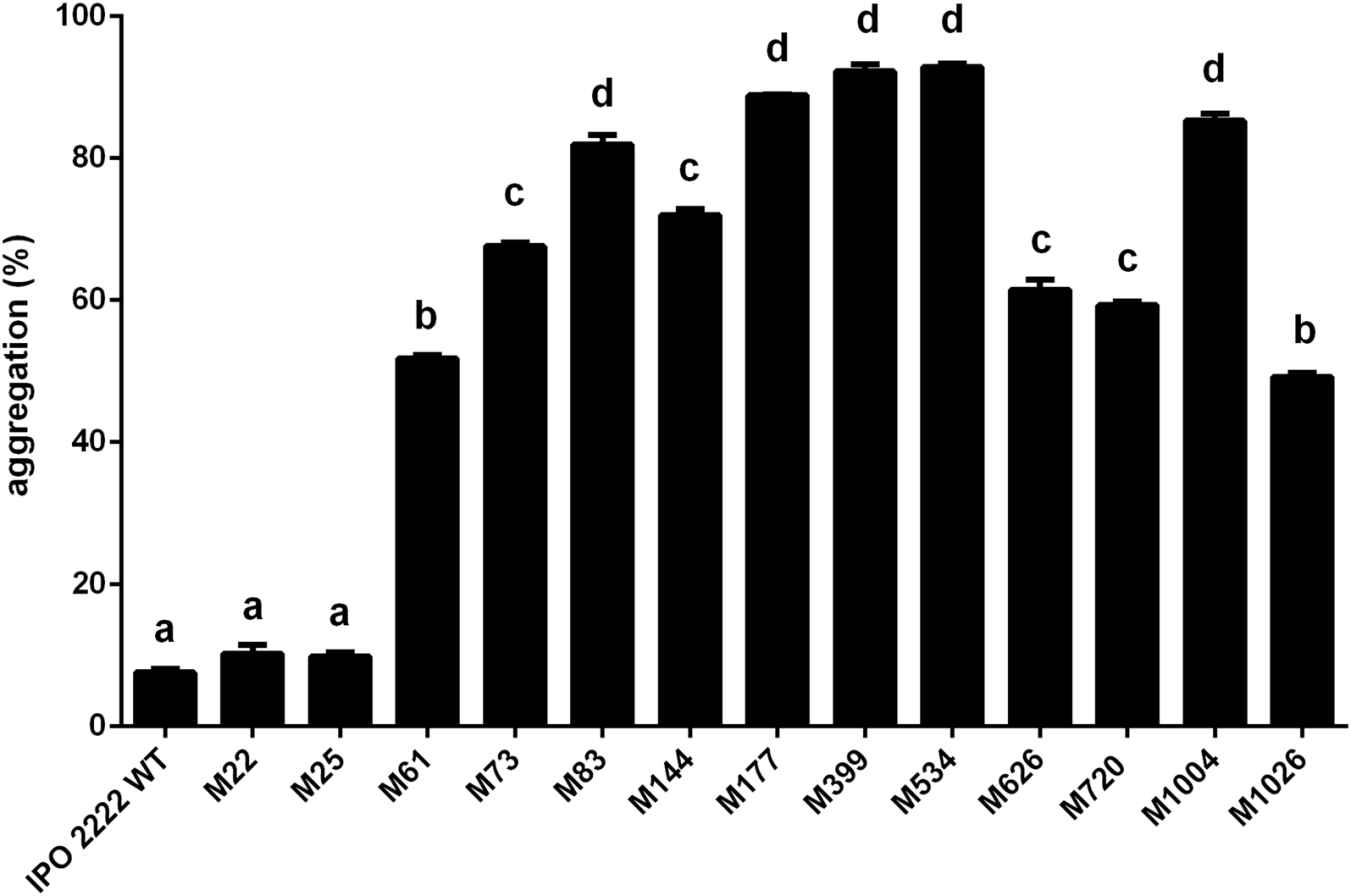
Self-aggregation of cells of WT and phage-resistant *D. solani* mutants as measured by turbidity of bacterial suspensions. The percentage of aggregation was quantified from the change in optical density (OD600) over 24 h. Percentage aggregation (sedimentation) was measured as follow: %A = 1 (OD600_24h_/OD600 _0 h_), where: %A—percentage of aggregation (sedimentation), OD600_0h_—OD of bacterial culture at time 0 h, OD600_24h_—OD of bacterial culture at time 24 h. Results were considered to be significant at p = 0.05 and pair-wise differences were obtained using the t-test. Error bars represent standard deviation (SD).

Since cell surface features were apparently altered in phage-resistant mutants, we assessed the self-association properties of the mutants as such a change would be expected to influence this process. All mutants except M22 and M25 exhibited more rapid sedimentation, associated with self-aggregation, than the WT strain (Fig. 5). The highest rate of sedimentation was observed in mutants M83, M177, M399, M534 and M1004 (Fig. 5).

**Figure 6:**
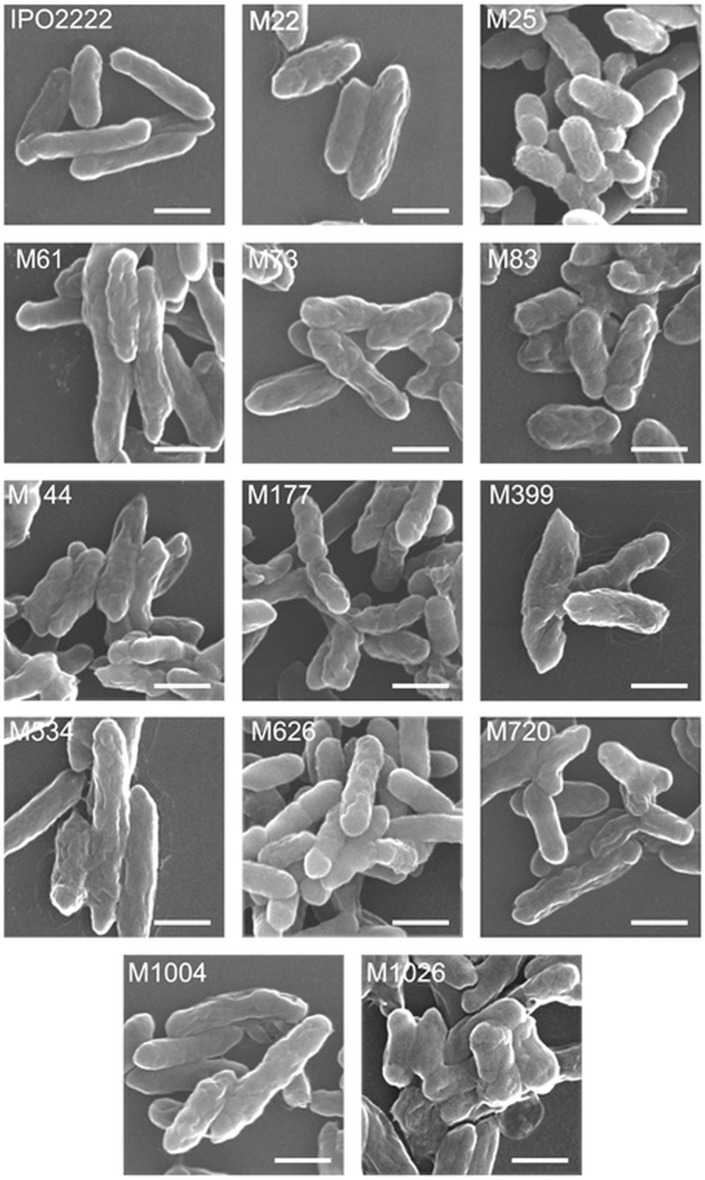
SEM imaging of *D. solani* WT and phage-resistant mutants. Scale bar corresponds to 1 µm.

As the differences in apparent self-aggregation suggested differences in surface features of at least some phage-resistant mutants we examined the surfaces directly with both SEM and Atomic Force Microscopy (AFM). While WT cells examined by SEM were characterized by a gently folded cell wall and rounded cell poles, all phage-resistant mutants had a rougher surface than the WT strain (Fig. 6). The degree of the cell wall folding in the mutants also differed from each other. Mutants M22 and M25 had a clearly spongy surface whereas mutants M61, M144, M534 and M1004 had longitudinal furrows on their surface (Fig. 6). On the other hand, mutants M73, M83, M177 and M626 were characterized by a lobular cell surface shape. Pointed cell poles and a longitudinal surface folding was specific to mutant M399 mutant. M720 cells, apart from having longitudinal furrows in the wall, were characterized by the presence of cell membrane forms bent even at an angle of 90°. Cells of mutant M1026 cells were clearly shorter than the other mutants and formed compact aggregates (Fig. 6).

**Figure 7:**
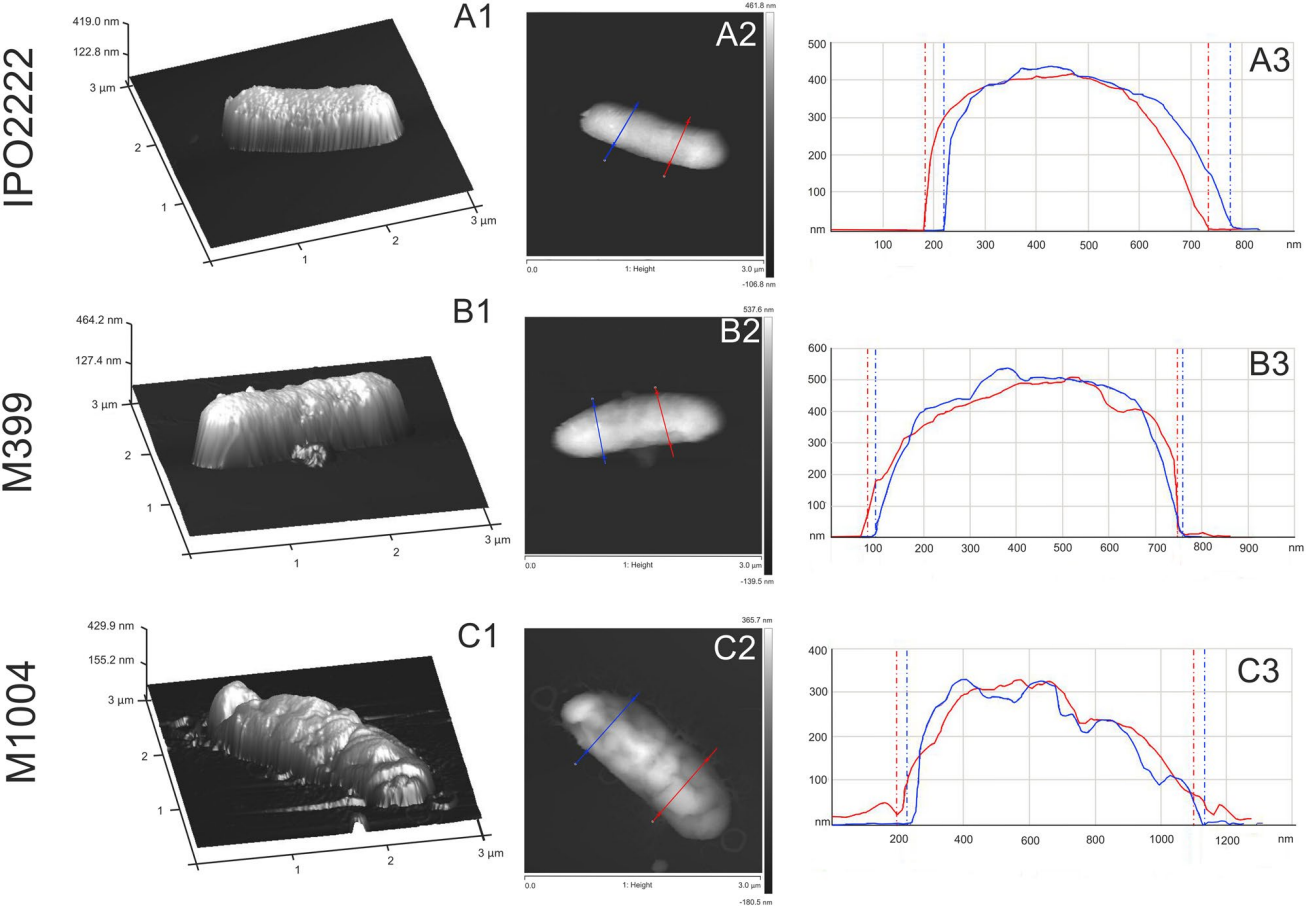
Atomic force microscopy (AFM) imaging of *D. solani* WT and selected phage-resistant mutants M399 and M1004. (panel A1–A3)—cell-surface *D. solani* IPO2222 WT; (panel B1–B3)—cell surface of phage-resistant mutant M399; (panel C1–C3)—cell surface of phage-resistant mutant M1004. (panel A1, B1, C1) –three-dimensional images of *D. solani* WT and mutant cells; (panel A2, B2, C2) –height mode images of cells; (panel A3, B3, C3)—height profiles of cells, the profiles were made along the lines shown in the height images.

Given the obvious differences in surface features seen in phage-resistant mutants by SEM we compared mutants M399 and M1004 with the WT strain by Atomic Force Microscopy (AFM) to provide more details of their distinctive surfaces. These mutants both possessed a rougher surface than the other strains. Mutant M399 had a less regular surface than the WT strain while mutant M1004, had a more folded surface than either the WT or mutant M399. AFM imaging of the WT strain showed a regular and symmetrical cell profile (Fig. 7). Slight folds characterized its cell surface both at three-dimensional image and height profiles, as shown in (Fig. 7, panels A1-A3). The topological features of mutants M399 and M1004 was quite different from that of the WT strain, having distinct folding, and an irregular surface with furrows visible in the height profiles (Fig. 7, panels B1-B3, C1-C3). Moreover, the surface roughness of the WT was lower (average roughness, Ra = 3.05 nm) than both mutant M399 (Ra = 4.41 nm) and M1004 (Ra = 4.89).

To compare the profile of membrane proteins present on the surface of phage-resistant mutants and the WT strain an intact cell MALDI technique was used. While all mutants exhibited similar proteins patterns as the WT strain, subtle differences in proteins content, presumably due to compensatory alterations in membrane structure were seen in some phage mutants (Supplementary Results, Supplementary Fig. 2).

### Survival of phage-resistant mutants on potato leaves

**Figure 8:**
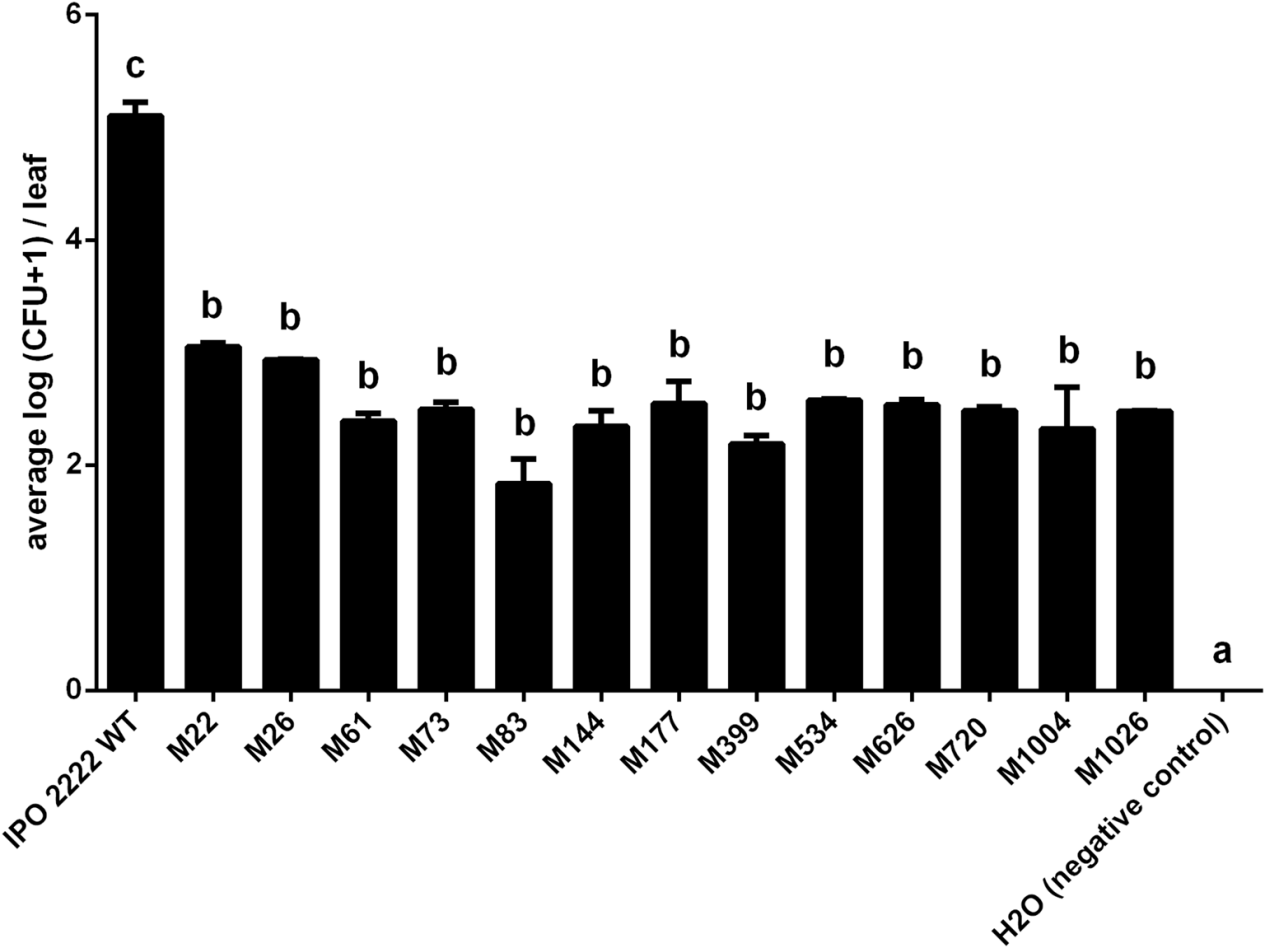
Population size of viable *D. solani* WT and phage-resistant mutants on the adaxial surface of detached leaves of *S. tuberosum* measured 14 days after inoculation. In replicated experiments, 20 leaves (10 leaves per experiment per mutant) were samples at two time points (0 [control] and 14 days post inoculation). Both at 0 and 14 dpi, five leaves (third to six from the shoot terminal) spray-inoculated with 2 ml of 10^6^ CFU mL^−1^ of bacterial suspension (WT or individual Tn5 mutant) in 1/4 Ringer’s buffer and incubated adaxial side up on 0.5% water agar in square plastic Petri dishes (100 × 100 mm) were sampled. At 0 and 14 dpi, five randomly chosen leaves from five randomly chosen Petri dishes were collected and individually shaken in 10 ml Ringer’s buffer in 50-mL Falcon tubes at 50 rpm at room temperature for 30 min to wash bacterial cells off the leaf surface. The serial diluted leaf washings were plated in duplicates on CVP supplemented with 200 µg mL^-1^ cycloheximide (for isolation of IPO 2222 WT) or on CVP containing 50 µg mL^-1^ neomycin ad 200 µg mL^-1^ cycloheximide (for isolation of phage-resistant Tn5 mutants). Inoculated plates were incubated at 28 °C for 24–48 h, and the resulting colonies were counted. The results were averaged for presentation. Results were considered to be significant at p = 0.05 and pair-wise differences were obtained using the t-test. Error bars represent standard deviation (SD).

Since *Dickeya* spp. may exist as an epiphyte on plant surfaces before invading the plant to cause disease^52^, any effect of phage resistance on epiphytic survival would be an important factor in their fitness as a pathogen under field conditions. The tolerance of stresses of the mutants on the adaxial surface of detached potato (*S. tuberosum*) leaf surfaces over a period of 14 days was thus investigated. (Fig. 8). As expected of such assays, the numbers of bacteria differed widely between leaves but relative differences between strains did not vary between replicate experiments, enabling the experiments to be analyzed together. Immediately after inoculation viable population sizes of 10^5^ to 10^6^ CFU per leaf for all strains were observed. No significant changes in the population size of the WT strain were observed over a 14-day incubation period (Fig. 8). In contrast, the viable population size of all phage-resistant mutants decreased significantly over this time to only ca. 10^2^–10^3^ CFU per leaf (Fig. 8). No pectinolytic, cavity-forming bacteria similar to *D. solani* were found on the leaves of non-inoculated leaves.

### Virulence of ΦD5-resistant mutants to potato

**Figure 9:**
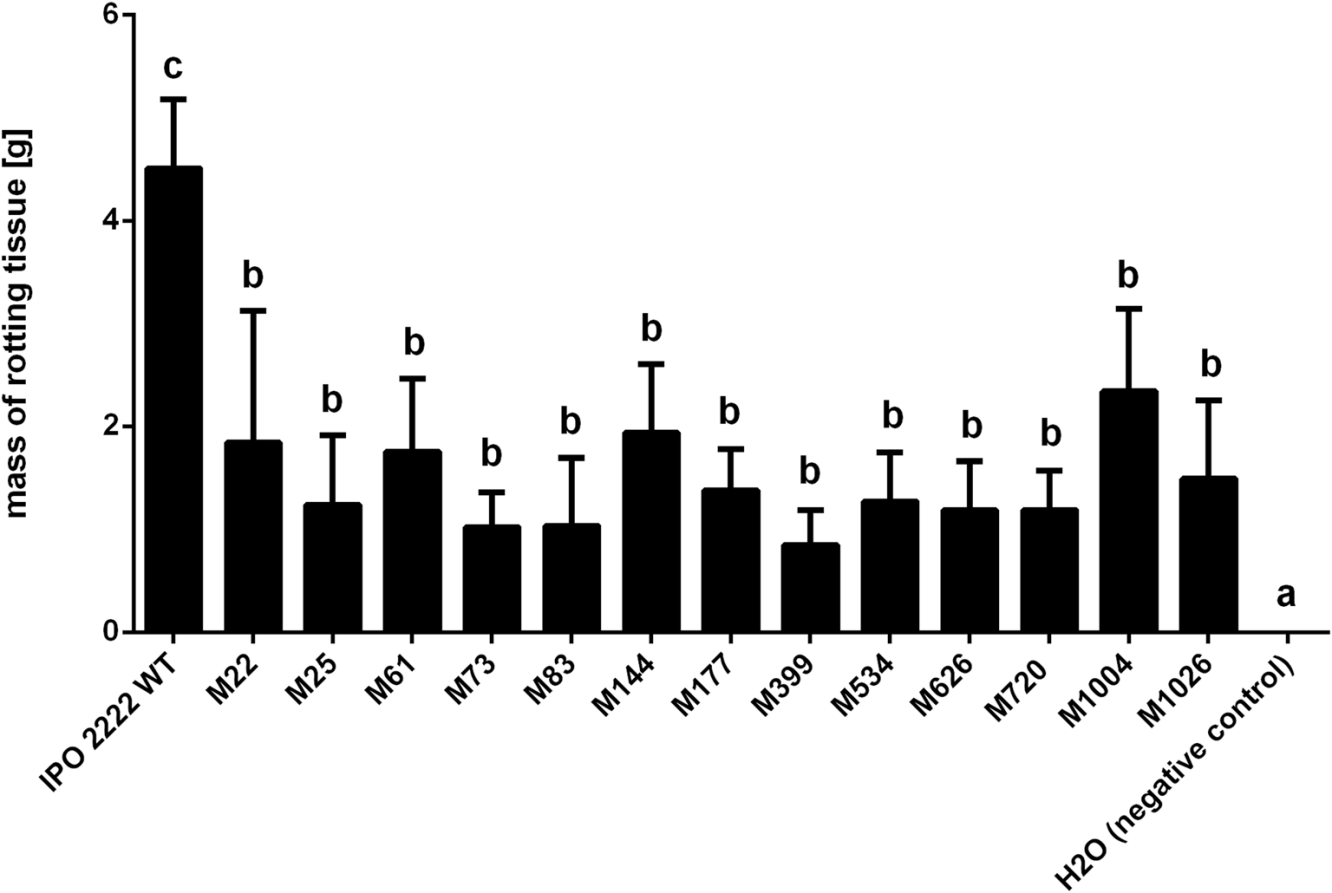
Maceration of potato tuber tissue by *D. solani* WT and phage-resistant mutants. Five individual potato tubers were inoculated with a given phage-resistant bacterial mutant using a whole tuber injection method (stab inoculation) ^103,106^. Bacterial strains were grown in TSB (WT strain), or TSB supplemented with 50 µg mL^-1^ of neomycin (Tn5 mutants) for 24 h at 28 °C. After incubation, bacterial cultures were separately collected, washed two times with 1/4 Ringer’s buffer and resuspended in the initial volume of Ringer’s buffer. Optical density (OD600 = 0.1) was used to normalize the number of bacterial cells in all treatments (ca. 10^8^ CFU mL^-1^). Surface-sterilized potato tubers were stab-inoculated with sterile yellow pipette tip filled with 50 µl of bacterial suspension (treatments) or sterile Ringer’s buffer (negative control). IPO 2222 WT was used as a positive control. Results were considered to be significant at p = 0.05 and pair-wise differences were obtained using the t-test. Error bars represent standard deviation (SD).

**Figure 10:**
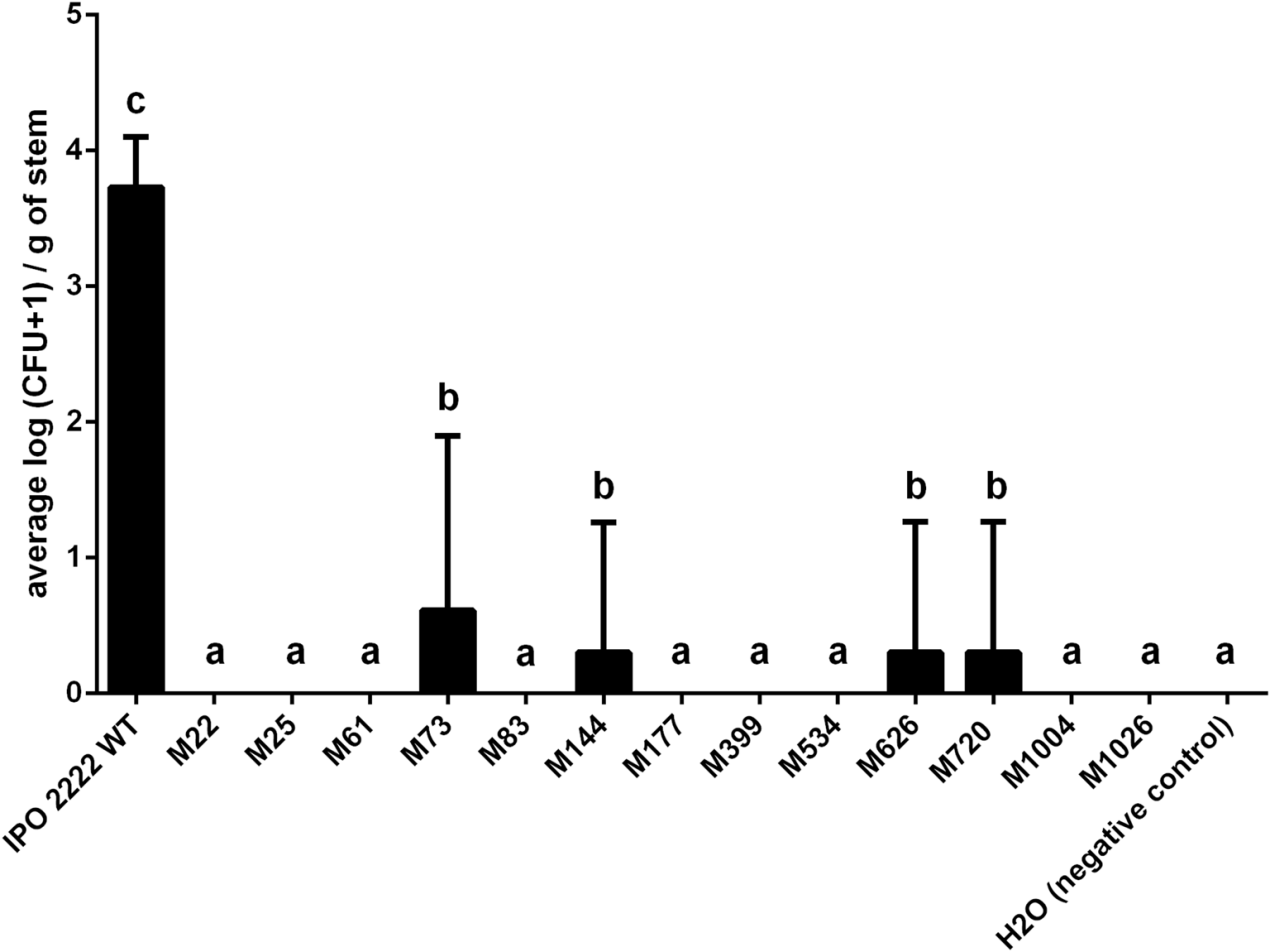
Population size of *D. solani* WT and phage-resistant mutants within stems of potato plants after introduction of the pathogen into the soil. Results were considered significant at *p* = 0.05, and the pair-wise differences were obtained using the t-test. The means that do not share the same letters above each bar differ. Results were considered to be significant at p = 0.05 and pair-wise differences were obtained using the t-test. Error bars represent standard deviation (SD).

Although all 13 phage-resistant mutants expressed some level of virulence, as measured by the degree of tissue maceration when introduced into the interior of potato tubers by stab-inoculation, they all were significantly affected in their ability to macerate potato tuber tissue compared with the WT strain (Fig. 9). Likewise, all mutants were significantly less virulent when tested for their ability to incite blackleg symptoms on potato plants grown in potting compost infested with bacteria (Fig. 10), a setting that reproduces the process by which the pathogen naturally invades the roots of growing plants from inoculum in the soil. Most potato plants inoculated with phage-resistant mutants in phytochamber experiments did not exhibit any blackleg symptoms. In contrast, between 80 and 100% of the plants inoculated with the WT strain developed severe and typical blackleg symptoms, which usually led to the death of some of the infected plants. As expected, no symptoms were observed at any time in plants inoculated with sterile buffer.

In addition to the large reduction in disease incidence incited by the mutants compared to the WT strain, the severity of infection, as measured by the number of viable bacterial cells within the tissue was also greatly reduced. Although the population size of the WT differed significantly between infected plants when measured 14 days after inoculation, it was recovered from all inoculated plants at densities ranging from 10^3^ to 15 × 10^3^ CFU g^−1^ of the stem tissue (Fig. 10). Phage-resistant mutants were recovered with both: (i) a much lower incidence and (ii) lower population size from stems after soil infestation. Among all phage mutants tested (10 plants per mutant), viable cells were detected in only 4 plants expressing typical blackleg symptoms; M73, M144, M626 and M720 were each recovered only once, and each with a very low population size (ca. 1000 CFU g^−1^ of stem tissue). None of the other mutants was detected inside stems of inoculated plants in both experiments (Fig. 10). As expected, *D. solani* was never detected in the stems of non-inoculated control plants.

### Competition of *D. solani* strains tagged with GFP or DsRed

The degree of competition of mutant and wild type strains, a sensitive method for comparing fitness differences, was assessed in different setting by quantifying changes in the proportion of the different strains in a mixture that differed in their color of fluorescence. The WT and phage-resistant mutants were transformed with plasmids pPROBE-AT-*gfp* and/or pRZ*-*T3*-dsred* conferring green and red fluorescence respectively. Both plasmids (Supplementary Table 1) were stably maintained in these strains as evidenced by maintenance on media without supplementation with ampicillin or tetracycline. Fluorescently-labelled bacterial variants displayed similar growth characteristics in liquid media to their parental strains (data not shown), indicating that their growth was not altered either by the presence of plasmids carrying *gfp* or *dsred* genes or by expression of fluorescent proteins in transformants.

**Figure 11:**
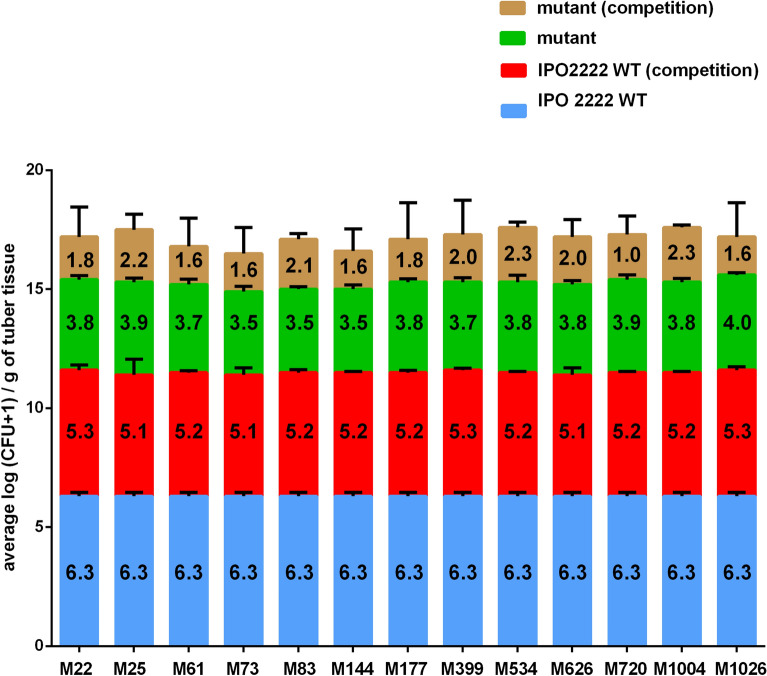
Population size of differentially marked cells of *D. solani* WT and phage-resistant mutants in potato tubers after co-inoculation or single inoculation into tubers. Potato tubers were inoculated either with fluorescently labelled *D. solani* IPO 2222 WT, fluorescently labelled individual phage-resistant *D. solani* Tn5 mutants or co-inoculated with WT strain and individual Tn5 mutant. In the first experiment, a GFP-tagged *D. solani* IPO 2222 WT and DsRed-tagged phage resistant *D. solani* mutants were used. In contrast, DsRed-tagged IPO 2222 WT and GFP-tagged phage resistant *D. solani* mutants were applied in the second experiment. Inoculated tubers were kept under conditions that promote rotting (28º and 80–90% relative humidity). Three days (72 h) post inoculation tubers were sampled and analyzed for the presence of fluorescently tagged bacteria using pour plating. The GFP and DsRed positive colonies were counted. The experiment was replicated one time with the same setup, and the results from both repetitions were averaged for analyses. The numbers inside bars represent the average log (CFU + 1/g of tuber tissue) for the respective treatment. Results were considered to be significant at p = 0.05 and pair-wise differences were obtained using the t-test. Error bars represent standard deviation (SD).

The competition of fluorescently-labelled WT and phage-resistant mutants co-inoculated into tubers by stabbing was assessed after 72 h incubation under conditions conducive for development of disease symptoms (28 °C, ca. 80–90% relative humidity (RH)) in phytochamber experiments (Fig. 11). The numbers of bacteria recovered differed among individual tubers but similar numbers were found for a given strain among replicate experiments and consequently, the results from both experiments were analyzed together. The population size of the WT strain inoculated alone into tubers reached between 10^6^ and 10^7^ CFU g^−1^ of tuber tissue by 72 h. Furthermore, the average populations of the WT strain co-inoculated with a phage-resistant mutant were only slightly reduced (to ca. 5 × 10^5^ CFU g^−1^) compared to that in controls inoculated only with the WT strain. The population size of any given mutant inoculated alone into tubers was greatly reduced (to ca. 10^3^–10^4^ CFU g^−1^) compared to that of the WT strain (Fig. 11). Importantly, the population sizes of any given mutant co-inoculated into tubers with the WT strain were greatly decreased (to ca. 10–5 × 10^2^ CFU g^−1^) compared to their already low population sizes achieved when inoculated alone into tubers (Fig. 11). These results strongly indicate that mutations that confer phage-resistance in *D. solani* impart severe decreases in its ability to infect plants.

## Discussion

The results of this study reveal that the cost of resistance of *D. solani* to lytic phage ΦD5 is strongly dependent on the environmental context. Although resistance to ΦD5 did not affect many of the phenotypes tested in vitro, all mutants were significantly impacted in their ability both to survive on the plant surface and to cause disease symptoms in either potato plants or tubers. Unfortunately, many details of the interactions of soft rot *Pectobacteria* such as *D. solani* and its host plant have not yet been revealed, so it is not possible to understand what aspects of its interaction with its host are most impacted by changes associated with phage resistance. Likewise, their interactions with lytic bacteriophages, especially the ecological context of such associations, remain insufficiently explored^53,54^. This study used a random Tn5-based transposon mutagenesis method to identify *D. solani* mutants resistant to infection caused by lytic phage ΦD5. The power of this approach was to unambiguously associate disruption of a given gene with changes in the behavior and features of the pathogen. By focusing on phenotypes likely to be involved in the ecological success of the pathogen in the plant environment and interrogating the mutant directly in these plant settings, this approach allowed us to investigate the tradeoff hypothesis in which the ΦD5 resistance confers fitness costs for the phage-resistant *D. solani* variants during infection.

Screening a collection of 1000 random Tn5 mutants allowed us to rather completely interrogate those genes and traits required for susceptibility to this phage. It was interesting to find that the 13 Tn5 insertional mutations conferring resistance to ΦD5 were located in only a small pool of 5 genes with multiple insertions in a given gene of interest. Furthermore, the disrupted genes are components of only two operons (Fig. 1, Table 1), both previously described as involved in the envelope status of Gram-negative bacteria, including bacteria belonging to *Enterobacteriaceae* and *Pectobacteriaceae* families^55^. Specifically, these operons participate in the biosynthesis of the core region of LPS together with production of the O-antigen of LPS in Gram-negative bacteria (Fig. 1)^51,56^. As no phage resistant-mutants analyzed in this study had insertions in other genes and operons, we believe that bacterial envelope components (viz. LPS, EPS and extracellular capsule (CPS)) encoded by the operons mentioned above are particularly essential for interaction of ΦD5 with *D. solani* IPO 2222. To gain more insight about the role of LPS in the context of phage resistance, we compared the lipopolysaccharides from phage-resistant mutants with that of the WT strain. Surprisingly, the characterization of LPSs by SDS-PAGE did not reveal apparent structural differences between the LPS of the WT strain and Tn5 mutants that could immediately explain the ΦD5-resistant phenotype suggesting that small structural changes not revealed in such an analysis were sufficient to disrupt interaction with the phage.

Modification of bacterial envelope is one of the most common strategies of phage evasion in Gram-negative bacteria^57,58^. To date, however, only limited data exist on how frequently such a mechanism is employed by plant pathogenic bacteria such as *D. solani* to prevent phage infection. In this study, phage adsorption was completely blocked in 11 of 13 phage-resistant mutants expressed a complete inhibition and was substantially reduced in 2 others. This observation cleanly connects phage adsorption with the nature of the *D. solani* envelope. In earlier studies in which we analyzed the interaction of *Pectobacterium parmentieri* with its lytic bacteriophage ΦA38, we found out that the phage requires intact LPS to infect its host and that alterations in LPS synthesis protected *P. parmentieri* from viral infection^59^. Similar observations linking phage-resistance and cell surface features have appeared for other SRP bacteria, including *P. atrosepticum* strain SCRI 1043, *P. carotovorum* strain Pcc27 and *P. brasiliense* strain F152 (PB29)^60–62^ but not for *D. solani* strain IPO 2222.

Most knowledge of the ecological functions of bacterial envelope components such as LPS, EPS and CPS polysaccharides, have come from studies of human and animal pathogenic bacteria^63,64^, microorganisms that encounter quite different environments in their life cycle than plant pathogenic bacteria. The highly variable and often stressful habitat in which plant pathogens commonly exist may make them particularly dependent on a suitable cell envelop. Unfortunately, the role of bacterial surface status in the interaction of plant pathogens and their plant hosts has received much less attention^65^. The gene encoding a glycosyltransferase that is linked to extracellular polysaccharide synthesis was disrupted in mutants M22 and M25. Glycosyltransferases are involved in LPS and EPS synthesis in plant pathogenic and plant beneficial bacteria^66,67^. Glycosyltransferase was reported to be required for EPS and LPS production in the plant pathogenic bacterium *Xanthomonas citri* subsp. *citri* (Xac). Furthermore, glycosyltransferase defective Xac mutants were impaired in biofilm and EPS production, exhibited delayed and reduced growth and were more susceptible to environmental stresses than the wild type strain^66^. In the plant-beneficial bacterium *Rhizobium* sp. YAS34, a polysaccharide-associated glycosyltransferase was involved in the synthesis of EPS, and was critical for colonization of roots as this function was required for cell adherence to roots during plant colonization^67^. Mutants M22 and M25 exhibited similar phenotypic defects in this study, having reduced growth in minimal medium in vitro, reduced synthesis of EPS, and decreased swimming and complete lack of swarming motility. The *cpsB* (*manC*) gene disrupted in mutants M61 and M1026 encodes a mannose-1-phosphate guanylyltransferase/mannose-6-phosphate isomerase. *cpsB* was reported to be required for virulence of *Streptococcus penumoniae* and *Klebsiella pneumoniae* in mice^68,69^. A homolog of *cpsB* is a part of the GDP-fucose synthesis pathway involved in the synthesis of a capsular polysaccharide colanic acid in the plant pathogenic bacterium *Erwinia amylovora*. Consequently, *E. amylovora* mutants lacking a functional *cpsB* were avirulent to its plant host^70^. Likewise, colanic acid was required for full virulence of a soft rot *Pectobacterium* spp. closely related to *Dickeya* spp.^71^. In *Xanthomonas campestris* pv. *campestris* (Xcc), a homolog of *cpsB,* gene *xanA* is required for the synthesis of extracellular polysaccharide xanthan, involved in stress tolerance and required by Xcc both for attachment to plant surfaces and biofilm formation^72^. Here, *D. solani* phage-resistant mutants M61 and M1026 with a disrupted locus encoding mannose-1-phosphate guanylyltransferase/mannose-6-phosphate isomerase exhibited elevated biofilm formation, reduced swimming motility and increased EPS production. The *wzt* (*rbfB*) gene was disrupted in *D. solani* mutants M73, M144, M626 and M720. This gene encodes the O-antigen export system (LPS export system) ATP binding protein involved in the translocation of O-antigen across the inner cell membrane to the periplasm, attaching it to a lipid A core^73^. In *Rhizobium tropici* CIAT899, a *wzt* deletion mutant had a lower growth rate, reduced motility, and lower colonization of roots than the wild type strain^74^. In *D. dadantii* A1828, *wzt* mutants had reduced survival of osmotic stress imposed by NaCl^75^. In the gliding bacterium *Myxococcus xanthus* blockage of its O-antigen export system ATP binding protein led to defects in social motility and colony formation under laboratory conditions^76^. In our study, all 4 *wzt* mutants shared similar phenotypes that were divergent from that of the WT strain including elevated EPS production, higher biofilm formation and reduced motility. *wbeA* encoding a hypothetical protein most closely related to the glycosyltransferase WbeA was disrupted in mutants M83, M399 and M534. The role of WbeA has not been assessed in plant pathogenic bacteria and the knowledge of its function in the ecology and pathogenicity of SRP bacteria is limited^51^. *wbeA* gene is a conserved member of the putative O-antigen LPS gene cluster present in various *Dickeya* species^51^. Its glycosyltransferase activity and genomic location suggest its involvement in LPS synthesis, together with another glycosyltransferase, WbeB, present in the same operon^51^. In our study, mutants M83, M399 and M534 had elevated biofilm formation, reduced EPS production and were incapable of swarming. Mutants M177 and M1004 had a mutation in *fcl* encoding GDP-L-fucose synthase. This enzyme is required for the production of the extracellular polysaccharide colanic acid in *Escherichia coli* strain K-12 and other members of *Enterobacteriaceae*^77^. Fcl was also reported to be involved in the synthesis of EPS in *Pectobacterium carotovorum*^78^. A *fcl* knockout mutant of *P. carotovorum* produced elevated biofilm but was less virulent to plants^78^. These phenotypes were similar to that seen here in phage-resistant mutants M177 and M1004.

To further support a role of surface features of *D. solani* for infection by ΦD5 we compared the surface of the WT and phage-resistant mutants using several techniques. While phage-resistant mutants were generally indistinguishable from the WT strain in overall the colony and cell morphology, more detailed analyses with SEM and AFM revealed that all had rougher and more irregular cell surfaces than the WT strain. In line with our observations, several other studies have showed that bacterial mutants with rough envelope phenotypes are more resistant to viral infection^79,80^. Furthermore, envelope regularity and smoothness resulting from LPS, EPS and capsule cooperation are also among the key factors determining the ability of bacterial to form biofilms on various surfaces and to survive in harsh environments^81,82^. Alterations of envelope properties may therefore increase antibiotic susceptibility, influence biofilm formation and reduce resistance to environmental stresses^83^. In our study, while all phage-resistant mutants did not differ from the WT in resistance to various antibiotics they did have elevated cell aggregation and biofilm formation phenotypes as well as higher membrane permeability than the WT strain.

All phage-resistant *D. solani* IPO 2222 mutants were compromised in their ability to cause disease symptoms *in planta*. Their decreased survival on the leaf surface and their low virulence in both potato tubers and growing potato plants were not due to a reduced growth rate of the mutants. The mutants and WT had similar generation times in rich media and only two mutants M22 and M25 had a slightly reduced growth in a minimal medium compared to the WT strain. The lower relative fitness of the phage-resistant mutants *in planta* was most clearly revealed in competition assays in which potato tubers were inoculated with mixtures of the WT strain and an individual phage-resistant mutant. Not only did all phage-resistant mutants achieve lower population sizes in potato than the WT strain when applied individually to potato tubers, but were particularly impaired in growing *in planta* when co-inoculated with the WT strain. Therefore, it is clear that the modifications of the cell envelope strongly impacts both the survival of *D. solani in planta* and its virulence. Interestingly, *wbeA* and *gmd*, the two members of the putative O-antigen LPS biosynthesis operon involved in interaction of *D. solani* with phage ΦD5, were also found to be induced in the presence of potato tuber tissue in our prior studies of this pathosystem^84^. As *gmd* and *wbeA,* together with *cpsB*, *cpsG*, *wzm*, *wzt*, *flc*, and *wbeB* constitute one operon (Fig. 1), we can assume that this operon is required both during infection of potato tubers as well as for infection of *D. solani* by ΦD5. It is thus clear that the bacterial envelope plays a central role both in the communication of *D. solani* with both their external world and its interaction with lytic bacteriophages. For this reason, alternations of the envelope to avoid viruses will likely impact their ecological fitness. The practical relevance of this observation is that by using phage therapy in agricultural applications (e.g. under field conditions or in greenhouses) we may be able not only to kill pathogens in situ but also to select for less virulent bacterial variants that under high and constant phage pressure may spread in the given niche and cause less problems in these environments in the future^85^.

Indeed, SRP bacteria, including *D. solani*, encounter harsh conditions inside the host during infection^86^. These include oxidative and osmotic stresses and exposure to antimicrobial compounds produced by plant injured tissues^87^. To cope with such stresses during infection, the bacterial envelope undergoes an extensive remodeling involving changes in LPS, EPS and capsule^87^. Mutations in the genes encoding envelope components in *D. solani* have been shown to lead to decreased survival and performance during infection^88^. Similar observations were made for other plant pathogenic bacteria. For example, envelope-defective mutants of *Ralstonia solanacearum* exhibit decreased virulence in tobacco plants^89^ and mutations altering envelope polysaccharides of *Erwinia amylovora* resulted in a reduced ability of the mutant to survive *in planta* and cause disease in pear^90^.

In conclusion, this study is one of few to investigate the tradeoff between phage resistance and fitness of bacterial plant pathogens in their natural environment. Likewise, this study is an initial step toward better understanding of how lytic bacteriophages interact with *Dickeya* spp., including *D. solani*. Using several complementary approaches, we showed that phage-resistant *D. solani* variants pay a substantial fitness penalty during their interaction with potato plants, suggesting that the resistance to phage ΦD5 strongly reduces its virulence. Furthermore, although these tradeoffs are linked with several modifications of the bacterial envelope, these costs are paid only *in planta* and not under in vitro conditions. This, in turn, suggests that fitness costs due to phage resistance in plant pathogenic bacteria is more frequently in nature than has been projected from in vitro studies.

## Methods

### Bacteriophages, bacterial strains, and growth media

The lytic bacteriophage vB_Dsol_D5 (ΦD5) was previously described^46,47,91^. For this work, ΦD5 was propagated on its wild-type host, *D. solani* strain IPO 2222^38^, and quantified as described earlier^46^. A stock of ΦD5 phage particles (ca. 10^8^—10^9^ plaque-forming units (PFU) mL^−1^ in tryptone soya broth (TSB, Oxoid) or quarter-strength (1/4) Ringer’s buffer (Merck) was used in all experiments unless otherwise stated. Bacterial strains used in this study are listed in Supplementary Table 1. The pool of 1000 mutants from a collection of 10,000 *D. solani* Tn5 mutants previously generated^84,92^ was interrogated as a source of ΦD5-resistant mutants. The *D. solani* wild type (WT) strain was cultivated for 24–48 h at 28 °C on tryptic soy agar (TSA, Oxoid), in tryptone soy broth (TSB, Oxoid) or M9 minimal medium (MP Biomedicals) supplemented with glucose (0.4%) (Sigma-Aldrich). Bacteriological agar (Oxoid) (15 g L^−1^) was added to solidify the media. As appropriate, media were supplemented with neomycin (Sigma-Aldrich) (50 µg mL^−1^), ampicillin (Sigma-Aldrich) (150 µg mL^−1^) or tetracycline (Sigma-Aldrich) (40 µg mL^−1^). Bacterial cultures were agitated during incubation (120 rpm). To prevent fungal growth, cycloheximide (Sigma-Aldrich) was added to the growth medium at a final concentration of 200 µg mL^−1^.

### Selection of ΦD5-resistant *D. solani* mutants

Phage-resistant *D. solani* Tn5 mutants were preselected as previously described^93^. Briefly, the overnight cultures of individual Tn5 mutants, grown in TSB supplemented with neomycin to the final concentration of 50 µg mL^−1^ and containing ca. 10^8^ colony-forming units (CFU) mL^−1^, were diluted 100-times in the same fresh medium (final bacterial density ca. 10^6^ CFU mL^−1^). Per Tn5 mutant to be analyzed, two wells of a 96-well plate were inoculated with 50 µl of individual IPO 2222 mutant culture (final bacterial density per well: ca. 5 × 10^4^ CFU). Subsequently, they were filled with 200 µl of ΦD5 solution (final phage concentration per well: ca. 2 × 10^7^ PFU). To each well of the microtiter plate, 5 µl of sterile 0.7% resazurin (Sigma) solution in demineralized water was added. After inoculation, 96-well plates were sealed with an optically transparent sealing type (Sarstedt) to prevent contamination and evaporation of bacterial cultures during incubation. The plates were incubated for 16 h with shaking (150 rpm) at 28 °C. After this time, the development of the pink/yellowish color (positive reaction) in the inoculated wells was determined by eye. The positive reaction indicated the growth of the respective Tn5 mutant in the presence of viable bacteriophages (= phage resistance). ΦD5-susceptible *D. solani* strain IPO 2222 and ΦD5-resistant *Escherichia coli* strain DH5α (ThermoFisher Scientific), grown under the same conditions and treated similarly as described above in the case of IPO 2222 Tn5 mutants, were used as controls^93^. Each Tn5 mutant was tested in four replicates for its resistance against ΦD5 phage. To confirm the resistance of the candidate Tn5 mutant to bacteriophage infection, each putative mutant obtained in the resazurin assay screen was additionally exposed to repeated phage challenge (at least 2 independent assays per mutant) and plaque formation assays (at least two independent assays per mutant) as previously described^46^.

### Identification of the transposon insertion sites in phage-resistant *D. solani* mutants

To identify the Tn5 insertion sites in the genomes of phage-resistant mutants, the genome of each mutant was sequenced. Genomic DNA of each mutant was isolated, sequenced, and assembled into a draft genome at the Laboratory of DNA Sequencing and Oligonucleotide Synthesis (Institute of Biochemistry and Biophysics of the Polish Academy of Science, Warsaw, Poland) using Illumina technology. Structural and functional annotations of the draft Tn5 genomes were determined using RAST (Rapid Annotation using Subsystem Technology (http://rast.nmpdr.org/).94The position of the Tn5 transposons in the draft genomes was established using BlastN and BlastX alignments accessed via the NCBI website (http://blast.ncbi.nlm.nih.gov/Blast.cgi)^95^. Using the available complete genome sequence of *D. solani* WT strain IPO 2222 (Genbank accession: CP015137)^45^ and the draft genomes of the Tn5 mutants the insertion sites was determined as described before^84^. For each mutant, sequences (ca. 1,000- to 5,000-bp) adjacent to the Tn5 transposon site were examined to determine the genomic context of each Tn5-disrupted gene^84,92^. The function of the disrupted genes was inferred using BlastN and BlastX alignments accessed via the NCBI website (https://blast.ncbi.nlm.nih.gov/Blast.cgi). Similarly, the functions of any unannotated open reading frames (ORFs) encoding hypothetical proteins or proteins without apparent sequence homology to proteins deposited in protein databases were inferred using GeneSilico Protein Structure Prediction meta-server^96^, together with PSI-BLAST (https://blast.ncbi.nlm.nih.gov/Blast.cgi) ^97^. The gene functions with the highest scores obtained were judged to be the most probable.

### Characterization of *D. solani* transcriptional units disrupted by the presence of Tn5 transposon

The putative transcriptional organization of *D. solani* genes interrupted by Tn5 was established using Operon-mapper (https://biocomputo.ibt.unam.mx/operon_mapper/). Inference of the biochemical pathways in which the genes of interest might participate was made using KEGG^98^. The results were visualized using iPath^99^. Likewise, proteins were evaluated for their predicted biological, functional, and metabolic roles in cellular networks using STRING (Search Tool for Retrieval of Interacting Genes/Proteins) v11.5 accessed via the website (https://string-db.org/) (parameters: network type: *full network*, network edges: *high confidence*, interaction sources: *text mining, experiments, databases, co-expression, co-occurrence, gene fusion*), providing information regarding interactions of proteins of interest^100^ using the proteome of the WT *D. solani* strain as a reference.

### Kinetics of ΦD5 adsorption to WT *D. solani* and Tn5 mutants

The rate of ΦD5 adsorption to wild-type *D. solani* and phage-resistant mutants was determined as before^59,101^. Briefly, log-phase bacterial cultures were inoculated with a phage suspension (Multiplicity of Infection (MOI) of 0.01) and incubated at 28 °C for up to 20 min. Two individual samples of each bacterial strain were collected after 0 (control), 1, 2, 5, 10, 15, and 20 min after inoculation with phage, and the number of unadsorbed phages was quantified. As a negative control, bacteriophages were suspended in sterile TSB and recovered at different times as above. The experiment was repeated three times, and the results were averaged. Phage adsorption efficiency was calculated as the average titer of unabsorbed phages per sample/average titer of phages in the negative control) x 100^59^.

### Kinetics of ΦD5 adsorption to killed *D. solani* cells

To test whether bacteriophage ΦD5 can adsorb to non‐viable cells of *D. solani*, a dead cell adsorption assay was employed^59,102^. Briefly, the WT strain was grown for 16 h in TSB at 28 °C with shaking and chloramphenicol (Sigma‐Aldrich, Darmstadt, Germany) was then added to a final concentration of 5 mg mL^−1^. The complete killing of the cells after 1 h was confirmed by plating 100 μL aliquots of treated bacterial culture on TSA plates. The chloramphenicol‐killed *D. solani* cells were mixed with a phage suspension (MOI = 0.01) and analyzed for phage adsorption as above. Three repetitions of the experiment were performed, and the results were averaged for analysis. Phage adsorption efficiency was calculated as described above.

### Determination of growth dynamics of phage-resistant Tn5 mutants of *D. solani* in rich and minimal media

To determine whether the Tn5 insertions affect the growth rate of the mutants, the growth of the selected *D. solani* Tn5 phage-resistant mutants was assessed both in TSB (rich medium) and in M9 containing 0.4% glucose (minimal medium) at 28 °C for 16 h as previously described^103^. The experiment was replicated once, and the results were averaged. The average generation time of each Tn5 mutant was analyzed using the Doubling Time calculator (parameters: C0 = 3 h, Ct = 7 h, t = 4 h) (http://www.doubling-time.com/compute.php) ^104^. The ability of ΦD5-resistant Tn5 mutants and the WT strain to grow was also tested qualitatively on solid rich and minimal media at temperatures: 4, 8, 15, 28, 37, and 42 °C as described before^105^. For this, 5-µL aliquots of 50-fold diluted in TSB or M9 + glucose overnight bacterial cultures grown in either TSA and M9 + glucose, respectively, were placed on the surface of either TSA or M9 + glucose and incubated for 120 h at 5 and 8 °C, or for 48 h at 15, 28, 37 and 42 °C. Growth was assessed visually daily. The experiment was replicated one time using the same setup.

To assess whether the transposon-mediated ΦD5-resistance differentially affected the growth rate of the *D. solani* mutants at different pHs, the growth of selected *D. solani* ΦD5-resistant Tn5 mutants was compared in TSB at pH 5 and pH 10, similarly to other studies^106^. Briefly, overnight cultures in TSB (ca. 10^9^ CFU ml^−1^) were diluted 50-fold in fresh growth broth with pH 5 or pH 10. Aliquots (100 µL of the diluted bacterial cultures) were aseptically transferred to the wells of 96-well microtiter plates and sealed with optically clear sealing tape (Sarstedt) to prevent evaporation. Bacterial growth rate was determined by measuring the optical density (λ = 600 nm) every 0.5 h for 12 h in an Epoch2 Microplate Spectrophotometer (BioTek). The experiment was repeated once, the generation time was calculated as above, and the results were averaged.

### Phenotypes of phage-resistant *D. solani* Tn5 mutants analyzed using BIOLOG Phenotypic Microarrays

The metabolism of phage-resistant *D. solani* mutants was analyzed in GEN III and EcoPlate microplates in a BIOLOG phenotypic microarray system (Biolog Inc.) as described before^59^. Inoculum was grown on TSA plates for 24 h at 28 °C, and then suspended in inoculation fluid (IF-A) (GENIII) or in 10 mM phosphate buffer pH 7.4 (EcoPlate) using a sterile cotton swab (Bionovo). The turbidity of the bacterial suspensions was adjusted to ca. 90% T with a spectrophotometer [A = log(%T)]. Bacterial suspensions (100 µl) were inoculated into duplicate wells of a 96-well microplate. The wells were then examined for a color change as evidence of growth and resource consumption. Color development was quantified using an Epoch2 microplate spectrophotometer (BioTek) equipped with a λ = 595-nm wavelength filter. Plates inoculated with the wild-type *D. solani* strain were used as controls.

### Phenotypes of phage-resistant *D. solani* Tn5 mutants

ΦD5-resistant *D. solani* mutants were screened for phenotypic features, putatively important for their interaction with plant tissues and/or environmental fitness, including swimming and swarming motility^106^, biofilm formation^107^, the ability to grow on TSA medium supplemented with 5% NaCl^108^, production of enzymes: pectinolytic enzymes^109^, cellulases^110^, proteases^111^ and siderophores^112^.

Phage‐resistant mutants were also interrogated by whole‐cell MALDI‐TOF MS spectral analysis as previously described^59^. Briefly, WT and mutants were grown on TSA at 28 °C for 24 h and then sampled with a loop and placed onto a MALDI plate. A mixture 1:1 (v/v) of ferulic acid (FA, 10 mg/ml in 33% acetonitrile, 13% formic acid, water) and dihydroxybenzoic acid (DHB, 10 mg/ml in 50% acetonitrile in water and 0.1% trifluoroacetic acid) was used as a matrix. 1 μL of matrix solution was used to overlay a bacterial spot, and the plate was then left to crystallize at room temperature for 15 min. Protein mass fingerprints were then obtained using a 5800 MALDI-TOF/TOF mass spectrometer (AB Sciex, Framingham, MA, USA), with detection in the linear middle mass in the range from 5000 to 20 000 Da, positive ion mode for a total of 1000 laser shots with a 1 kHz OptiBeam laser (YAG, 349 nm)^59^. The MS spectra obtained were examined with Data Explorer Software (AB Sciex). All MALDI‐TOF MS spectra reported were averages of six replicated measurements (2 independent measurements of each of 3 technical repetitions) for each strain analyzed as described before^59^.

### Measurement of morphological features of cells and colonies

The colony morphology of phage-resistant mutants was analyzed using a Leica MZ10 F stereomicroscope with 10 × and 40 × magnifications coupled to a Leica DFC450C camera (Leica) as previously described^59,92^.

The cell morphology of phage resistant mutants was evaluated using transmission electron microscopy (TEM) as previously described^103^. TEM analyses were performed at the Laboratory of Electron Microscopy (Faculty of Biology, University of Gdansk, Poland). Bacteria were adsorbed onto carbon-coated grids (GF Microsystems), directly stained with 1.5% uranyl acetate (Sigma-Aldrich), and visualized with an electron microscope (Tecnai Spirit BioTWIN, FEI) as described previously^103^. At least ten images of each mutant and the wild-type strain were taken to estimate cell diameter.

WT and phage-resistant mutant cells were fixed for Scanning Electron Microscopic analyses using a standard procedure^113^. Briefly, bacterial cells were fixed with 4% glutaraldehyde in 0.1 M phosphate buffer, pH 7.0, treated with OsO4, dehydrated in a graded series of acetone concentrations (15%, 30%, 50%, 70% and twice 100%), followed by drying the specimens using silica gel for 24 h, The samples were then gold-sputtered using a K550X sputter coater (Quorum Technologies) and visualized with a Vega 3 Scanning Electron Microscope (Tescan)^113^.

Detailed imaging of the surface of selected strain cells was done using atomic force microscopy (AFM) as previously described^113^. The measurements were taken in Peak-Force Quantitative Nanomechanical Mapping mode using a NanoScope V AFM (Bruker, Vecco Instruments Inc., Billerica, MA, USA) equipped with NanoScope 8.15 software. The force constant of the NSG01 probe (NT-MDT Spectrum Instruments, Russia) was in the range 1.45–15.1 N/m. Three fields of 3 µm × 3 µm were scanned for each sample. The roughness was read from 10 areas of 100 nm × 100 nm from each image, and the arithmetic mean was calculated from the obtained results. Images were analyzed with Nanoscope Analysis version 1.4.

### Autoaggregation of Tn5 mutants

The autoaggregation of strains was assessed as the degree of accelerated sedimentation and phase separation of aqueous bacterial suspensions^114^. Phage-resistant mutants were assessed for the speed of autoaggregation (sedimentation) as described in^115,116^. Briefly, WT (control) and phage-resistant mutants were grown in 10 ml TSB at 28 °C for 24 h with shaking and then aliquoted (1 ml) transferred to sterile cuvettes (Eppendorf), and the optical density (OD600) was measured initially (time = 0) and again after cuvettes (covered with parafilm) were incubated for 24 h at 28 °C without shaking. Two replicates were done per each bacterial strain and the experiment was repeated once. The results from both repetitions were averaged for analysis. Percentage aggregation (sedimentation) was measured as follow: %A = 1-(OD600_24h_/OD600 _0 h_), where: %A—percentage of aggregation (sedimentation), OD600_0h_—OD of bacterial culture at time 0 h, OD600_24h_—OD of bacterial culture at time 24 h^115^.

### Permeability of cell outer membrane

Permeability of cellular outer membranes was assessed by the degree of cell lysis observed following exposure of bacteria to sodium dodecyl sulphate (SDS)^117^. Briefly, cells were grown in TSB (WT strain) or in TSB supplemented with 50 μg mL^−1^ neomycin (Tn5 mutants) for 16 h at 28 °C with shaking. After incubation 2 ml of culture was washed two times with PBS pH 7.2 (Sigma-Aldrich) and resuspended in 2 ml of PBS to an OD600 = 0.1(ca. 10^8^ CFU mL^−1^). Aliquots (180 µL) were then transferred to wells of a 96-well plate (Greiner Bio-One) and either 20 µL of sterile water (control) or 10% SDS (Sigma-Aldrich) was then added. Cell lysis was monitored at room temperature via a decrease of optical density (OD) of the bacterial culture measured every minute for 10 min using an Epoch2 Microplate Spectrophotometer (BioTek). Two replicates were done for each bacterial strain and the entire experiment was repeated once. The results were averaged for analysis.

### Antibiotic susceptibility of Tn5 mutants

The antibiotic susceptibility of *D. solani* mutants was determined with a disc diffusion method as previously described^59,118^. Antibiotic discs (BD BBL—Sensi-Disc antimicrobial test discs) containing chloramphenicol (30 µg), gentamicin (10 µg), tigecycline (15 µg), doxycycline (30 µg), sulfametoxazol/trimetropin (23,75/1.25 µg), ciprofloxacin (5 µg), ceftaroline (5 µg), imipenem (10 µg), piperacillin/tazobactam (30/6 µg), cefuroksym/ceftaroline (30/5 µg), cefuroxime (30 µg), aztreonam (30 µg), ampicillin (10 µg), ampicillin/sulbactam (10/10 µg), colistin (10 µg), fosfomycin (200 µg) were applied to bacterial seeded plates of Mueller–Hinton (MH medium, BD) supplemented with 1.5% agar (Oxoid). Inoculum of phage-resistant mutants grown for 16 h in TSB supplemented with neomycin (50 μg mL^−1^) or WT grown in TSB at 28 °C with shaking (120 rpm). The plates were inoculated using a sterile cotton swab soaked in a bacterial suspension and the antibiotic discs were placed on the agar surface in such a way to ensure a minimum distance between each disc of ca. 1.5—2 cm. Plates were incubated at 28 °C for 24 h and examined for the presence of a clear halo surrounding a given disc. The experiment was repeated once.

### Isolation and visualization of lipopolysaccharide (LPS) from wild-type *D. solani* strain IPO 2222 and phage-resistant *D. solani* Tn5 mutants

Lipopolysaccharides (LPS) of bacterial strains were isolated using a Lipopolysaccharide Extraction Kit (Abcam, Symbios, Gdansk, Poland) with a modified protocol^59^. Lipopolysaccharides were separated using 4–20% sodium dodecyl sulfate‐polyacrylamide gradient gel Mini‐PROTEAN® TGX™ Precast Protein Gel, BioRad, Hercules, USA) electrophoresis (SDS‐PAGE) according to^119^ and visualized with silver staining as described before^120^.

### Population dynamics of *D. solani* phage-resistant mutants on adaxial leaf surfaces of S. tuberosum

Potato plants *(S. tuberosum)* cultivar Kondor, cultivated as described earlier^47^, were grown in a growth chamber at 22 °C and 16/8 h (day/night) photoperiod (white cool fluorescent light, Philips, TLD 58 W/84o, 30–35 μmol m^−2^ s^−1^) and 80% relative humidity (RH). Leaves (third to sixth from the shoot tip) were detached from the plants and inoculated with bacteria as described earlier^121^. Briefly, bacterial suspensions (2 ml) adjusted to 10^6^ CFU mL^−1^ in sterile Ringer’s buffer (Merck) were sprayed onto the adaxial surface of each detached leaf using a manual sprayer. The moisture on sprayed leaves were then allowed to dry in a laminar flow hood and placed, adaxial side up, on 0.5% water agar (Oxoid) supplemented with 200 µg mL^−1^ cycloheximide (Sigma-Aldrich), to prevent fungal growth. Sterile Ringer’s buffer was used instead of bacterial suspensions as a control. The detached, inoculated leaves were incubated on the agar plates in the growth chamber under the same conditions used to grow the plants. Samples were collected immediately after placement in the growth chamber and after 14 days and assayed for the presence of viable *D. solani*. At each sample time 5 randomly chosen leaves were collected for a given strain and shaken in 10 ml of Ringer’s buffer in 50 ml Falcon tubes at 50 rpm at room temperature for 30 min to remove bacterial cells from the surfaces of the leaves. The leaf washings were then serially diluted in Ringer’s buffer and 100 μl of appropriate dilutions plated in duplicate on CVP supplemented with 200 μg ml^−1^ cycloheximide (Sigma) or on CVP containing 50 μg ml^−1^ neomycin and 200 μg ml^−1^ cycloheximide as appropriate. The inoculated plates were incubated at 28 °C and cavity-forming *D. solani* colonies enumerated. The experiment was replicated once, and the results were averaged.

### The ability of selected phage-resistant mutants to cause maceration of potato tubers

Potato tubers (5 replicate tubers per bacterial strain) of cv. Bryza purchased locally in Gdansk, Poland and selected for their uniform size (diameter of ca. 5–6 cm and weight of ca. 50 -70 g) were inoculated with a given strain and assessed for subsequent disease symptoms, using a whole tuber injection method (stab inoculation), as described before^103,106^. Bacterial suspension (ca. 10^8^ CFU mL^−1^) (100 µl per strain/site) was delivered to potato tuber by stab inoculation into tuber pith using a 200 µl pipette tip. Wild‐type *D. solani* was used as a positive control, and negative control plants were inoculated with sterile demineralized water. The experiment was repeated once.

### Virulence of *D. solani* Tn5 mutants in potato plants

Replicated experiments of plants grown in a growth chamber were performed in November and December 2021 using the previously developed protocol^47^. Certified potato tubers (cv. Kondor) were acquired from the Plant Breeding and Acclimatization Institute—National Research Institute, Bonin, Poland. *S. tuberosum* potato plants were cultivated in a growth chamber at 22 °C and 16/8 h (day/night) photoperiod (white cool fluorescent light, Philips, TLD 58 W/84o, 30–35 μmol m^−2^ s^−1^) and 80% relative humidity (RH) as described earlier^47^. After two weeks of cultivation, rooted plants with a height of ca. 10–15 cm were transferred to 1 L pots and cultivated in potting soil for 2 additional weeks under similar conditions. Potato plants (5 plants per replication) were inoculated with bacterial strains by the application of 50 ml of bacterial suspensions (10^8^ CFU mL^−1^) in sterile 1/4 Ringer’s buffer) directly to the soil surrounding stems 1 h after plants had been well watered to ensure uniformly moist soil. As a negative control, soil was treated with only sterile Ringer’s buffer (50 ml per plant). Pots were then randomized within a growth chamber with 5 blocks of 15 pots per treatment. Plants were visually inspected daily for the development of disease symptoms consisting of chlorosis, black rotting of the stem, haulm wilting, and the death of the plants. Plants were sampled 14 days post-inoculation by excising ca. 2 cm long stem segments, located ca. 5 cm above ground level, and pooled per analyzed plant^59^. The stem fragments were surface-sterilized as described before^47^. The presence of *D. solani* cells inside potato stems was determined by plating stem macerates on CVP supplemented with 200 μg mL^−1^ cycloheximide (WT strain) or CVP supplemented with 50 μg mL^−1^ neomycin and 200 μg mL^−1^ cycloheximide (Tn5 mutants) and counting the resulting bacterial colonies. The experiment was replicated once, and the results were averaged for analysis.

### Competition of bacterial strains on potato tubers

#### Generation of fluorescently-tagged strains

Plasmids pPROBE-AT-*gfp*^122^ and pRZ*-*T3*-dsred*^123^, used previously for stable tagging of *D. solani* cells with fluorescent proteins^124,125^, were used to generate GFP- and dsRed-tagged variants of phage-resistant *D. solani* mutants and/or WT strain, respectively. Plasmids pPROBE-AT-*gfp* and pRZ*-*T3*-dsred* were introduced into bacterial cells by electroporation as described in^125^. After electroporation, bacterial cultures (100 µL) were plated on TSA plates containing 100 µg mL^−1^ ampicillin or 40 µg mL^−1^ tetracycline as appropriate and incubated for 24–48 h at 28 °C. The GFP and dsRed-tagged mutant strains were grown on TSA supplemented with neomycin (50 µg mL^−1^) and additionally supplemented with ampicillin (100 µg mL^−1^) for selection of GFP-tagged strains or tetracycline (40 µg mL^−1^) for selection of DsRed-tagged *D. solani* strains unless stated otherwise. About 20 to 50 transformants were recovered per strain obtained per transformation event. One bacterial colony expressing a high fluorescent signal was collected and used for further analysis in each case. In addition, the growth rate of fluorescent protein-tagged strains was evaluated as described above.

#### Co-inoculation of potato tubers with fluorescently tagged WT and ΦD5-resistant mutants

To determine whether phage-resistant mutants were altered in their fitness during infection of potato tubers compared to the WT strain, two independent tuber co-inoculation experiments were conducted. In the first experiment, a GFP-tagged WT (IPO 2254)^125^ strain together with DsRed-tagged phage-resistant Tn5 *D. solani* mutants were used, whereas in the second experiment, DsRed-tagged WT and GFP-tagged phage-resistant mutants were employed. The tagged WT strain was grown in TSB supplemented with ampicillin (100 µg mL^−1^: GFP-tagged) or tetracycline (40 µg mL^−1^: DsRed-tagged), respectively for 16 h at 28 °C with shaking (200 rpm). Phage-resistant Tn5 mutants tagged either with GFP or DsRed were grown under the same conditions, but the medium was additionally supplemented with neomycin (Sigma-Aldrich) to a final concentration of 50 µg mL^−1^. Bacterial cultures were collected by centrifugation (6000 × RCF, 5 min.), washed twice with sterile 1/4 Ringer’s buffer and resuspended in sterile Ringer’s buffer to a density of ca. 10^8^ CFU mL^−1^. Potato tubers of cultivar Bryza (5 replicate tubers/strain), selected for their uniform size (diameter of ca. 5–6 cm and weight of ca. 50 -70 g) that were obtained locally in Gdansk, Poland (and shown to be free of *Pectobacterium* spp. and *Dickeya* spp. as described earlier^126^) were rinsed with running tap water to remove soil particles, surface-sterilized for 20 min in 5% commercial bleach solution in water, and washed twice for 1 min with demineralized, sterile water. Surface-sterilized tubers were then dried in a laminar flow hood. Tubers were injected with bacterial strains as described earlier^103,106^. A volume of 100 µl of bacterial culture was delivered to each tuber by stab inoculation of 100 µl bacterial suspension with a pipette tip as above. Treatments included: (i): 10^6^ CFU mL^−1^ of WT strain, (ii): 5 × 10^5^ CFU mL^−1^ of WT together with 5 × 10^5^ CFU mL^−1^ of a phage-resistant mutant or (iii): 10^6^ CFU mL^−1^ of a phage-resistant mutant alone. Inoculated tubers were kept in humid boxes (80–90% relative humidity) at 28 °C for 72 h to allow bacteria to rot potato tissue. After incubation, ca. 2 g of rotted potato tissue was collected from each tuber and resuspended in 2 ml Ringer’s buffer/g of tissue^126^. To protect bacterial cells from oxidative stress, the Ringer’s buffer was supplemented with the anti-oxidant, 0.02% diethyldithiocarbamic acid (DIECA, Sigma-Aldrich). Aliquots (100 µL of appropriate dilutions of tissue macerates were mixed with 300 µL of PT medium^109^ prewarmed to 45–50 °C and containing 200 µg mL^-1^ cycloheximide, 50 µg mL^-1^ neomycin and either 150 µg mL^-1^ ampicillin (for selection of the GFP-tagged strains) or 40 µg mL^-1^ tetracycline (for selection of the DsRed-tagged strains) in wells of a 48-well plate (Greiner Bio-One). Plates containing solidified medium were incubated for 24 to 48 h at 28 °C. Wells were inspected for the presence of GFP- and DsRed-fluorescent bacterial cells using an epifluorescence stereomicroscope (Leica MZ10 F and Leica DFC450C camera system)^127^ and GFP-positive and DsRed-positive colonies were counted. The experiment was replicated once, and the results were averaged for analysis.

### Statistical analyses

Statistical analyzes were performed as previously described^59^. Briefly, bacterial colony counts were transformed as log(x + 1) to achieve normality. Each time, the treatments were analyzed matching the experimental pattern in which two replicated experiments were done per each treatment of replicated plants/tubers/leaves. The Shapiro–Wilk test (p < 0.05)^128^ was used for those samples where the distribution of bacterial population size was described by a normal distribution. For those samples not normally distributed (e.g. control vs. treatment or treatment vs. treatment), the Welch’s T‐test was applied^129^. The variance homogeneity was validated using the Fisher–Snedecor test^130^. Pair‐wise differences were evaluated using a two‐tailed Student’s t‐test^131^. The linear model was a complete block design with replicates as individual blocks^132^. The main effects observed were analyzed for the impact of time and treatment type and a two‐way interaction between time and treatment type.

## Supplementary Information

Supplementary Information.

## Data Availability

Data generated or analyzed during this study are included in this published article (including its Supplementary Information files). In addition, the raw genome sequences of the *D. solani* Tn5 phage-resistant mutants are deposited at Zenodo (www.zenodo.org) under https://doi.org/10.5281/zenodo.6587967, protein mass fingerprints (MALDI-TOF/TOF) are deposited at Zenodo under https://doi.org/10.5281/zenodo.6588037 and all TEM, SEM and AFM raw photos and accompanying data are deposited at Zenodo under https://doi.org/10.5281/zenodo.6587895.
