# Supplementary material for: Resistance of Dickeya solani strain IPO 2222 to lytic bacteriophage ΦD5 results in fitness tradeoffs for the bacterium during infection
Source: Sci Rep. 2022 Jun 24;12:10725. doi: 10.1038/s41598-022-14956-7 (PMC9232599; doi:10.1038/s41598-022-14956-7)
Supplement: Supplementary file 1 — Supplementary Information. [file 41598_2022_14956_MOESM1_ESM.pdf]

Resistance of *Dickeya solani* strain IPO 2222 to lytic bacteriophage ΦD5 results in fitness tradeoffs for the bacterium during infection

Przemyslaw Bartnik [1], Kinga Lewtak [2], Marta Fiołka [3], Paulina Czaplewska [4], Magdalena Narajczyk [5] and Robert Czajkowski [1] *

[1] Laboratory of Biologically Active Compounds, Intercollegiate Faculty of Biotechnology UG and MUG, University of Gdansk, Antoniego Abrahama 58, 80-307 Gdansk, Poland

[2] Department of Cell Biology, Institute of Biological Sciences, Maria Curie-Sklodowska University, Akademicka 19, 20-033 Lublin, Poland

[3] Department of Immunobiology, Institute of Biological Sciences, Maria Curie-Sklodowska University, Akademicka 19, 20-033 Lublin, Poland

[4] Laboratory of Mass Spectrometry-Core Facility Laboratories, Intercollegiate Faculty of Biotechnology UG and MUG, University of Gdansk, Antoniego Abrahama 58, 80-307 Gdansk, Poland

[5] Laboratory of Electron Microscopy, Faculty of Biology, University of Gdansk, Wita Stwosza 59, 80-308 Gdansk, Poland

*** Correspondence:**

Robert Czajkowski, robert.czajkowski@ug.edu.pl

Supplementary Information

Supplementary Results

Characterization of surface proteins with mass spectrometry (MS)

For all mutants, except for M61, the pattern in the m / z from the 6000 to 6600 range was similar. The most intense signals at 9534.6 m/z and two distinct ones at 6522.1 and 7203.2 m / z are visible in each of them as well. One can see changes between the intensity of the last two mentioned signals, but there is no regularity in the analyzed groups (one of them prevails in the group in each mutant). However, subtle differences can be found between individual groups or single variants versus wild-type cells. For example, the M22 and M25 variants have clearly distinguished two signals at 7266.9 and 7287.2 m / z, which have a much lower intensity (Supplementary Figure 1). Similarly, intense signals are distinguished for variants M177, M83 and M626. In all cases, proteins corresponding to signals above 10,000 m/z are hardly visible. Most of them are broad, low-intensity signals. Most variants present fewer peaks in this range than the wild variant. The signal at 10665.2 is present in every MS spectrum. The preceding 10,300 m / z signal seen by IPO2222 disappears in the remaining cells. It is visible only for variants from the M83/399/534 group. They also give relatively distinct remaining higher masses between 15,000 to 17,000. Of the remaining variants, the peak at 16,222.7 is higher for M61 and M177. In the range of about 12,000 m/z, two signals, 12 290.2 and 12345.0, are visible for the WT while only the first signal is visible for most mutants.

Supplementary Tables

Supplementary Table 1. Bacterial strains used in this study and their relevant characteristics

No.	Strain	Origin	Relevant characteristics	Reference
1	**IPO 2222**	potato, 2007, the Netherlands	Wild type (WT)	(van der Wolf et al., 2014)
2	**IPO 2254**	IPO 2222	pPROBE-AT-*gfp* (Miller et al., 2000), *amp*^{R}	(Czajkowski et al., 2010)
3	**IPO 2222-dsRed**	IPO 2222	pRZ-T3-*dsred* (Bloemberg et al., 2000), *tet*^{R}	This study
4	**M22**	IPO 2222	mini-Tn5-*gusA* (Xi et al., 1999), *neo*^{R}	This study
5	**M22-GFP**	M22	mini-Tn5-*gusA*, *neo*^{R}, pPROBE-AT-*gfp, amp*^{R}	This study
6	**M22-DsRed**	M22	mini-Tn5-*gusA*, *neo*^{R}, pRZ-T3-*dsred*, *tet*^{R}	This study
7	**M25**	IPO 2222	mini-Tn5-*gusA*, *neo*^{R}	This study
8	**M25-GFP**	M25	mini-Tn5-*gusA*, *neo*^{R}, pPROBE-AT-*gfp, amp*^{R}	This study
9	**M25-DsRed**	M25	mini-Tn5-*gusA*, *neo*^{R}, pRZ-T3-*dsred*, *tet*^{R}	This study
10	**M61**	IPO 2222	mini-Tn5-*gusA*, *neo*^{R}	This study
11	**M61-GFP**	M61	mini-Tn5-*gusA*, *neo*^{R}, pPROBE-AT-*gfp, amp*^{R}	This study
12	**M61-DsRed**	M61	mini-Tn5-*gusA*, *neo*^{R}, pRZ-T3-*dsred*, *tet*^{R}	This study
13	**M73**	IPO 2222	mini-Tn5-*gusA*, *neo*^{R}	This study
14	**M73-GFP**	M73	mini-Tn5-*gusA*, *neo*^{R}, pPROBE-AT-*gfp, amp*^{R}	This study
15	**M73-DsRed**	M73	mini-Tn5-*gusA*, *neo*^{R}, pRZ-T3-*dsred*, *tet*^{R}	This study
16	**M83**	IPO 2222	mini-Tn5-*gusA*, *neo*^{R}	This study
17	**M83-GFP**	M83	mini-Tn5-*gusA*, *neo*^{R}, pPROBE-AT-*gfp, amp*^{R}	This study
18	**M83-DsRed**	M83	mini-Tn5-*gusA*, *neo*^{R}, pRZ-T3-*dsred*, tet^R	This study
19	**M144**	IPO 2222	mini-Tn5-*gusA*, *neo*^{R}	This study
20	**M144-GFP**	M144	mini-Tn5-*gusA*, *neo*^{R}, pPROBE-AT-*gfp, amp*^{R}	This study
21	**M144-DsRed**	M144	mini-Tn5-*gusA*, *neo*^{R}, pRZ-T3-*dsred*, *tet*^{R}	This study
22	**M177**	IPO 2222	mini-Tn5-*gusA*, *neo*^{R}	This study
23	**M177-GFP**	M177	mini-Tn5-*gusA*, *neo*^{R}, pPROBE-AT-*gfp, amp*^{R}	This study
24	**M177-DsRed**	M177	mini-Tn5-*gusA*, *neo*^{R}, pRZ-T3-*dsred*, *tet*^{R}	This study
25	**M399**	IPO 2222	mini-Tn5-*gusA*, *neo*^{R}	This study

**26**	**M399-GFP**	M399	mini-Tn5-*gusA*, *neo*^{R}, pPROBE-AT-*gfp, amp*^{R}	This study
**27**	**M399-DsRed**	M399	mini-Tn5-*gusA*, *neo*^{R}, pRZ-T3-*dsred*, *tet*^{R}	This study
**28**	**M534**	IPO 2222	mini-Tn5-*gusA*, *neo*^{R}	This study
**29**	**M534-GFP**	M534	mini-Tn5-*gusA*, *neo*^{R}, pPROBE-AT-*gfp, amp*^{R}	This study
**30**	**M534-DsRed**	M534	mini-Tn5-*gusA*, *neo*^{R}, pRZ-T3-*dsred*, *tet*^{R}	This study
**31**	**M626**	IPO 2222	mini-Tn5-*gusA*, *neo*^{R}	This study
**32**	**M626-GFP**	M626	mini-Tn5-*gusA*, *neo*^{R}, pPROBE-AT-*gfp, amp*^{R}	This study
**33**	**M626-DsRed**	M626	mini-Tn5-*gusA*, *neo*^{R}, pRZ-T3-*dsred*, *tet*^{R}	This study
**34**	**M720**	IPO 2222	mini-Tn5-*gusA*, *neo*^{R}	This study
**35**	**M720-GFP**	M720	mini-Tn5-*gusA*, *neo*^{R}, pPROBE-AT-*gfp, amp*^{R}	This study
**36**	**M720-DsRed**	M720	mini-Tn5-*gusA*, *neo*^{R}, pRZ-T3-*dsred*, *tet*^{R}	This study
**37**	**M1004**	IPO 2222	mini-Tn5-*gusA*, *neo*^{R}	This study
**38**	**M1004-GFP**	M1004	mini-Tn5-*gusA*, *neo*^{R}, pPROBE-AT-*gfp, amp*^{R}	This study
**39**	**M1004-DsRed**	M1004	mini-Tn5-*gusA*, *neo*^{R}, pRZ-T3-*dsred*, *tet*^{R}	This study
**40**	**M1026**	IPO 2222	mini-Tn5-*gusA*, *neo*^{R}	This study
**41**	**M1026-GFP**	M1026	mini-Tn5-*gusA*, *neo*^{R}, pPROBE-AT-*gfp, amp*^{R}	This study
**42**	**M1026-DsRed**	M1026	mini-Tn5-*gusA*, *neo*^{R}, pRZ-T3-*dsred*, *tet*^{R}	This study

Supplementary Table 2. Predicted molecular functions of the *D. solani* IPO 2222 WT proteins involved in the interaction of the bacterium with phage vB_Dsol_D5 (ΦD5)

No	Insertion name, Tn5 locus	Localization in the *D. solani* IPO 2222 WT genome (Genbank accession: CP015137.1)	Homologs in *Dickeya* spp. [A]	Putative protein interaction partners according to STRING (https://string-db.org/) [B]
**1**	***p22, p25,* A4U42_09910, ANE75629.1**	2 344 388 – 2 345 530 (positive strand)	*D. dadantii,* *D. fangzhongdai,* *D. dianthicola,* *D. zeae,* *D. oryzae,* *D. undicola,* *D. chrysanthemi*	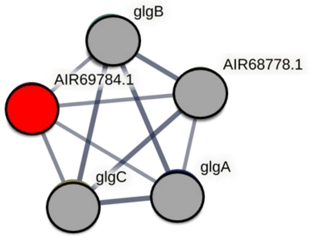<br>***glgA***- glycogen synthase; synthesizes alpha-1,4-glucan chains using ADP-glucose<br><br>***glgB*** – 1,4-alpha-glucan branching enzyme GlgB; catalyzes the formation of the alpha-1,6-glucosidic linkages in glycogen by scission of a 1,4-alpha-linked oligosaccharide from growing alpha-1,4-glucan chains and the subsequent attachment of the oligosaccharide to the alpha-1,6 position<br><br>***glgC*** - glucose-1-phosphate adenylyltransferase; Involved in the biosynthesis of ADP-glucose, a building block required for the elongation reactions to produce glycogen, catalyzes the reaction between ATP and alpha-D-glucose 1-phosphate (G1P) to produce pyrophosphate and ADP-Glc, belongs to the bacterial/plant glucose-1-phosphate adenylyltransferase family

				AIR68778.1 – phosphoglucomutase catalyzes the interconversion of alpha-D-glucose 1-phosphate to alpha-D-glucose 6-phosphate;
**2**	***p61, p1026 cpsB*, (*manC*) A4U42_06115, ANE74940.1**	1 442 004 – 1 443 401 (positive strand)	*D. dadantii*<br>*D. dianthicola,*<br>*D. fangzhongdai,*	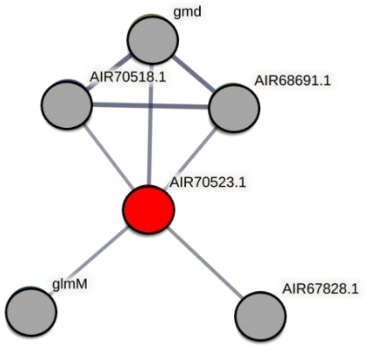<br><br>***gmd*** – GDP-mannose 4,6-dehydratase; Catalyzes the conversion of GDP-D-mannose to GDP-4-dehydro-6- deoxy-D-mannose.<br><br>***AIR70518.1*** – epimerase domain-containing protein<br><br>***AIR68691.1*** – UDP-glucose 6-dehydrogenase catalyzes the formation of UDP-glucuronate from UDP-glucose<br><br>***glmM*** – phosphoglucosamine mutase catalyzes the conversion of glucosamine-6-phosphate to glucosamine-1-phosphate, which belongs to the phosphohexose mutase family.<br><br>***AIR67828.1*** - PTS system, mannose-specific IID component, phosphoenolpyruvate-dependent sugar phosphotransferase system catalyzes the phosphorylation of incoming sugar substrates concomitant with their translocation across the cell membrane; IID with IIC forms the translocation channel

**3**	***p73, p144, p626, p720***<br>***wzt*, (*rbfB*)**<br>**A4U42_06135, ANE74944.1**	1 446 795 – 1 447 526 (positive strand)	*D. dianthicola,*<br>*D. fangzhongdai,*<br>*D. dadantii,*<br>*D. chrysanthemi,*<br>*D. oryzae,*<br>*D. undicola*	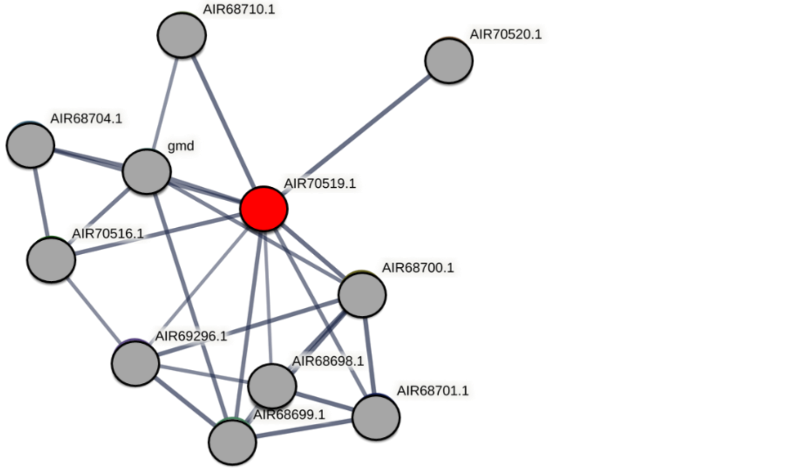<br><br>***AIR68710.1*** - polysaccharide export lipoprotein Wza, required for the translocation of capsular polysaccharide through the outer membrane<br><br>***AIR70520.1*** - ABC2 membrane domain-containing protein;<br><br>***AIR68704.1*** - mannosyltransferase;<br><br>***gmd*** – GDP-mannose 4,6-dehydratase, catalyzes the conversion of GDP-D-mannose to GDP-4-dehydro-6- deoxy-D-mannose<br><br>*AIR70516.1* – glycosyltransferase<br><br>***AIR68700.1*** – glucose-1-phosphate thymidylyltransferase, catalyzes the formation of dTDP-glucose, from dTTP and glucose 1-phosphate, as well as its pyrophosphorolysis<br><br>***AIR69296.1*** – UDP-N-acetylglucosamine 4,6-dehydratase

				AIR68698.1 – dTDP-4-dehydrorhamnose reductase, catalyzes the reduction of dTDP-6-deoxy-L-lyxo-4-hexulose to yield dTDP-L-rhamnose, belongs to the dTDP-4-dehydrorhamnose reductase family<br><br>***AIR68699.1*** – dTDP-4-dehydrorhamnose 3,5-epimerase, catalyzes the epimerization of the C3' and C5'positions of dTDP-6-deoxy-D-xylo-4-hexulose, forming dTDP-6-deoxy-L-lyxo-4-hexulose, belongs to the dTDP-4-dehydrorhamnose 3,5-epimerase family<br><br>***AIR68701.1*** - dTDP-glucose 4,6-dehydratase
**4**	***p83, p399, p534, wbeA*, A4U42_06145, ANE74946.1**	1 448 472 – 1 449 671 (positive strand)	*D. fangzhongdai, D. dadantii, D. dianthicola, D. zeae, D. oryzae*	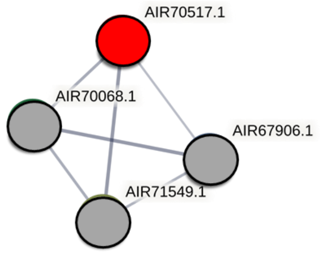<br><br>***AIR70068.1*** – maltose/maltodextrin ABC transporter, substrate binding periplasmic protein MalE<br><br>***AIR67906.1*** – putative ABC transporter, periplasmic substrate X binding protein;<br><br>***AIR71549.1*** - putative carbamoyl-phosphate-synthetase protein

5	***p177, p1004*** ***fcl,*** **A4U42_06140,** **ANE74945.1**	1 447 537 – 1 448 475 (positive strand)	*D. fangzhongdai,* *D. dadantii,* *D. dianthicola,* *D. zeae,* *D. oryzae,* *D. undicola,* *D. chrysanthemi*	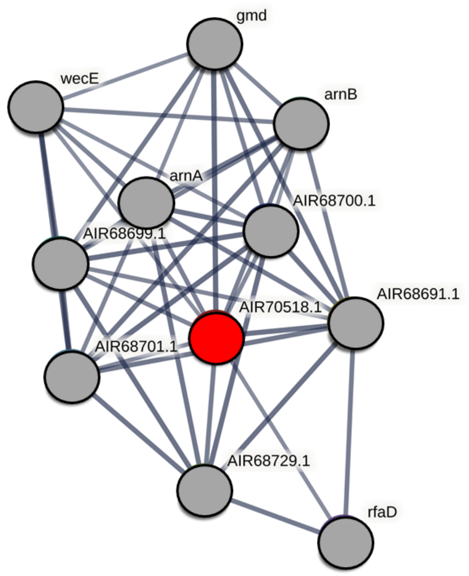 ***gmd*** – GDP-mannose 4,6-dehydratase, catalyzes the conversion of GDP-D-mannose to GDP-4-dehydro-6- deoxy-D-mannose<br><br>***wecE*** - dTDP-4-amino-4,6-dideoxygalactose transaminase, catalyzes the synthesis of dTDP-4-amino-4,6-dideoxy-D-galactose (dTDP-Fuc4N) from dTDP-4-keto-6-deoxy-D-glucose (dTDP-D- Glc4O) and L-glutamate, belongs to the DegT/DnrJ/EryC1 family<br><br>***arnB*** – UDP-4-amino-4-deoxy-L-arabinose--oxoglutarate aminotransferase; Catalyzes the conversion of UDP-4-keto-arabinose (UDP-Ara4O) to UDP-4-amino-4-deoxy-L-arabinose (UDP-L-Ara4N) (the modified arabinose is attached to lipid A and is required for resistance to polymyxin and cationic antimicrobial peptides), belongs to the DegT/DnrJ/EryC1 family, ArnB subfamily

				arnA – UDP-glucuronic acid oxidase, UDP-4-keto-hexauronic acid decarboxylating, bifunctional enzyme that catalyzes the oxidative decarboxylation of UDP-glucuronic acid (UDP-GlcUA) to UDP-4-keto- arabinose (UDP-Ara4O) and the addition of a formyl group to UDP-4- amino-4-deoxy-L-arabinose (UDP-L-Ara4N) to form UDP-L-4-formamido-arabinose (UDP-L-Ara4FN) (the modified arabinose is attached to lipid A and is required for resistance to polymyxin and cationic antimicrobial peptides), in the N-terminal section; belongs to the Fmt family, UDP- L-Ara4N formyltransferase subfamily<br><br>***AIR68699.1*** – dTDP-4-dehydrorhamnose 3,5-epimerase, catalyzes the epimerization of the C3' and C5'positions of dTDP-6-deoxy-D-xylo-4-hexulose, forming dTDP-6-deoxy-L-lyxo-4-hexulose, belongs to the dTDP-4-dehydrorhamnose 3,5-epimerase family<br><br>***AIR68700.1*** – glucose-1-phosphate thymidylyltransferase, catalyzes the formation of dTDP-glucose, from dTTP and glucose 1-phosphate, as well as its pyrophosphorolysis<br><br>***AIR68701.1*** – dTDP-glucose 4,6-dehydratase, belongs to the NAD(P)-dependent epimerase/dehydratase family, dTDP-glucose dehydratase subfamily.<br><br>***AIR68691.1*** – UDP-glucose 6-dehydrogenase, catalyzes the formation of UDP-glucuronate from UDP-glucose<br><br>***AIR68729.1*** – UDP-glucose 4-epimerase<br><br>***rfaD*** - ADP-L-glycero-D-manno-heptose-6-epimerase; Catalyzes the interconversion between ADP-D-glycero-beta-D-manno-heptose and ADP-L-glycero-beta-D-manno-heptose via an epimerization at carbon 6 of the heptose, belongs to the

				NAD(P)-dependent epimerase/dehydratase family, HldD subfamily

[A] – Search done with NCBI BlastP (https://blast.ncbi.nlm.nih.gov/Blast.cgi) using amino acid sequences of the protein from *D. solani* IPO 2222 WT, cutoff was set to 70% protein identity

[B] – Search Tool for Retrieval of Interacting Genes/Proteins v11.5 accessed *via* https://string-db.org/. *D. solani* IPO 2222 WT was used as a model to assess the putative interaction partners of bacterial proteins found in this study. Only high confidence (0.700 and above) scores are shown (Szklarczyk et al., 2019), line density between the nodes indicate the strength of data support, the protein of interest in marked in red circle and its interaction partners are marked with grey circles

Supplementary Figures

Supplementary Figure 1. Characterization of lipopolysaccharide (LPS) isolated from WT and phage-resistant *D. solani* mutants.

SDS-PAGE was made using a gradient (4-20%) polyacrylamide gel (Biorad), and the LPS components were visualized using silver staining. The size marker (11-245 kDa, Perfect Tricolor Protein Ladder, EURx, Poland) is shown in the first lane of both gels.

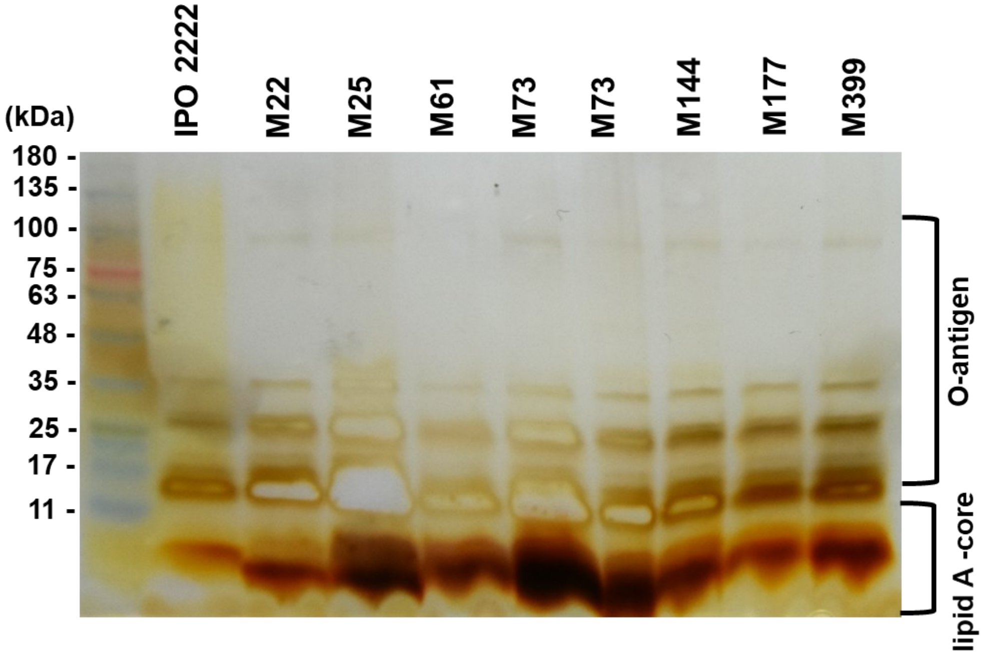

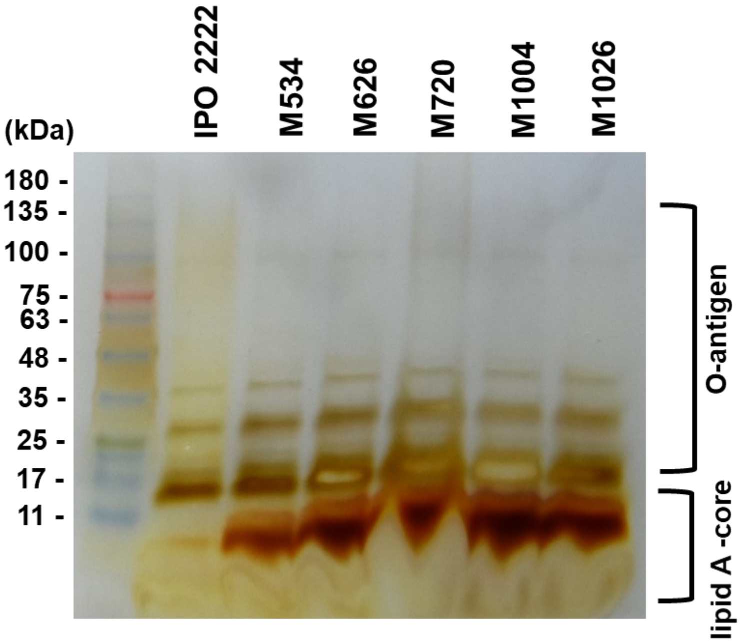

Supplementary Figure 2. Representative intact MALDI-TOF spectra of D. solani IPO 2222 WT and 13 phage-resistant mutants in a range from 5000 to 9400 m/z. The recorded spectra are organized according to Tn5 mutated loci: panel A) mutants M22 and M25; panel B) mutants M61 and M1026; panel C) mutants M73, M144, M626 and M720; panel D) mutants M83, M399 and M534 and panel E) mutants M177 and M1004. Average from two independent biological replicates, each containing three technical replicates are shown.

A)

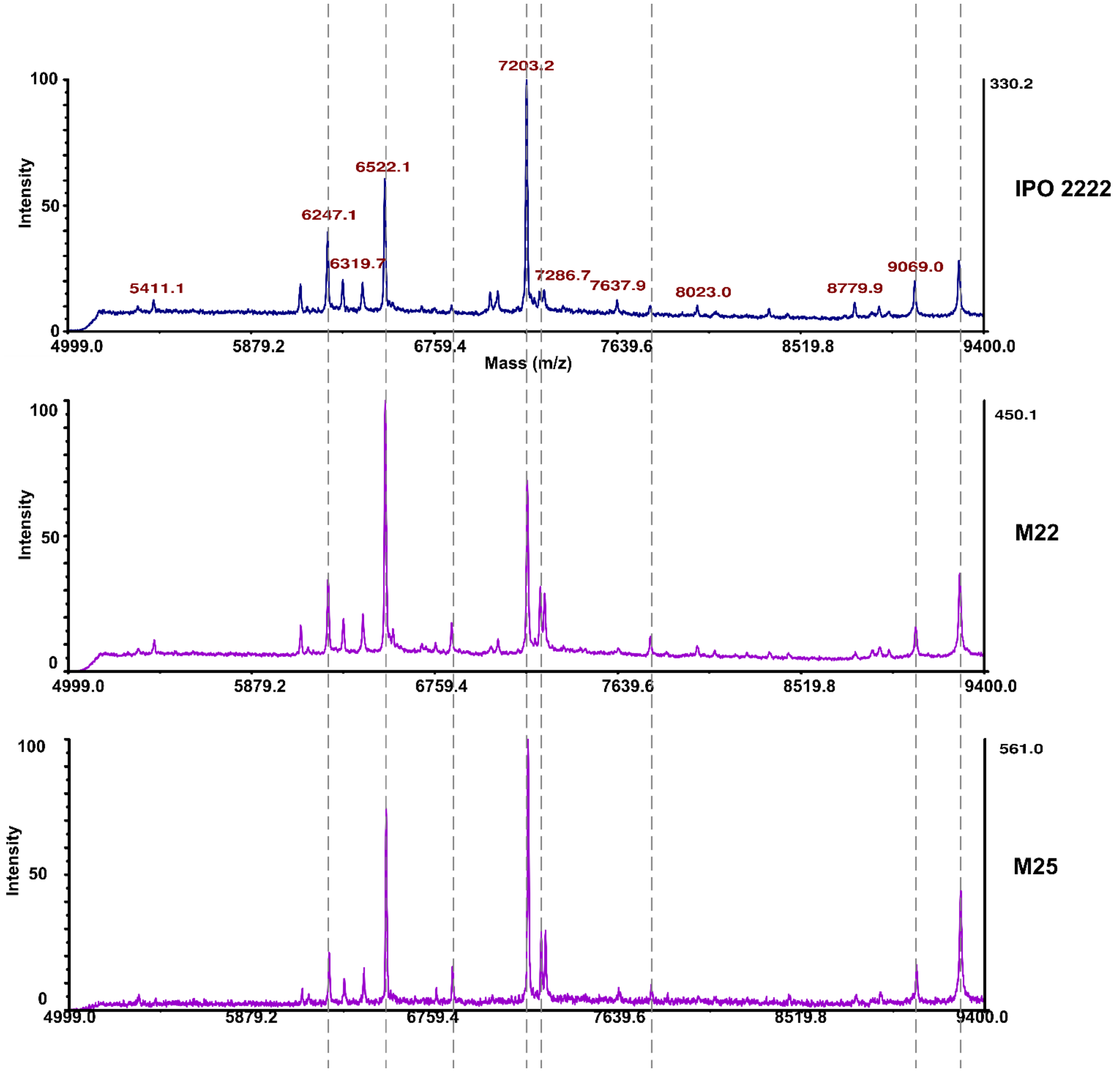

B)

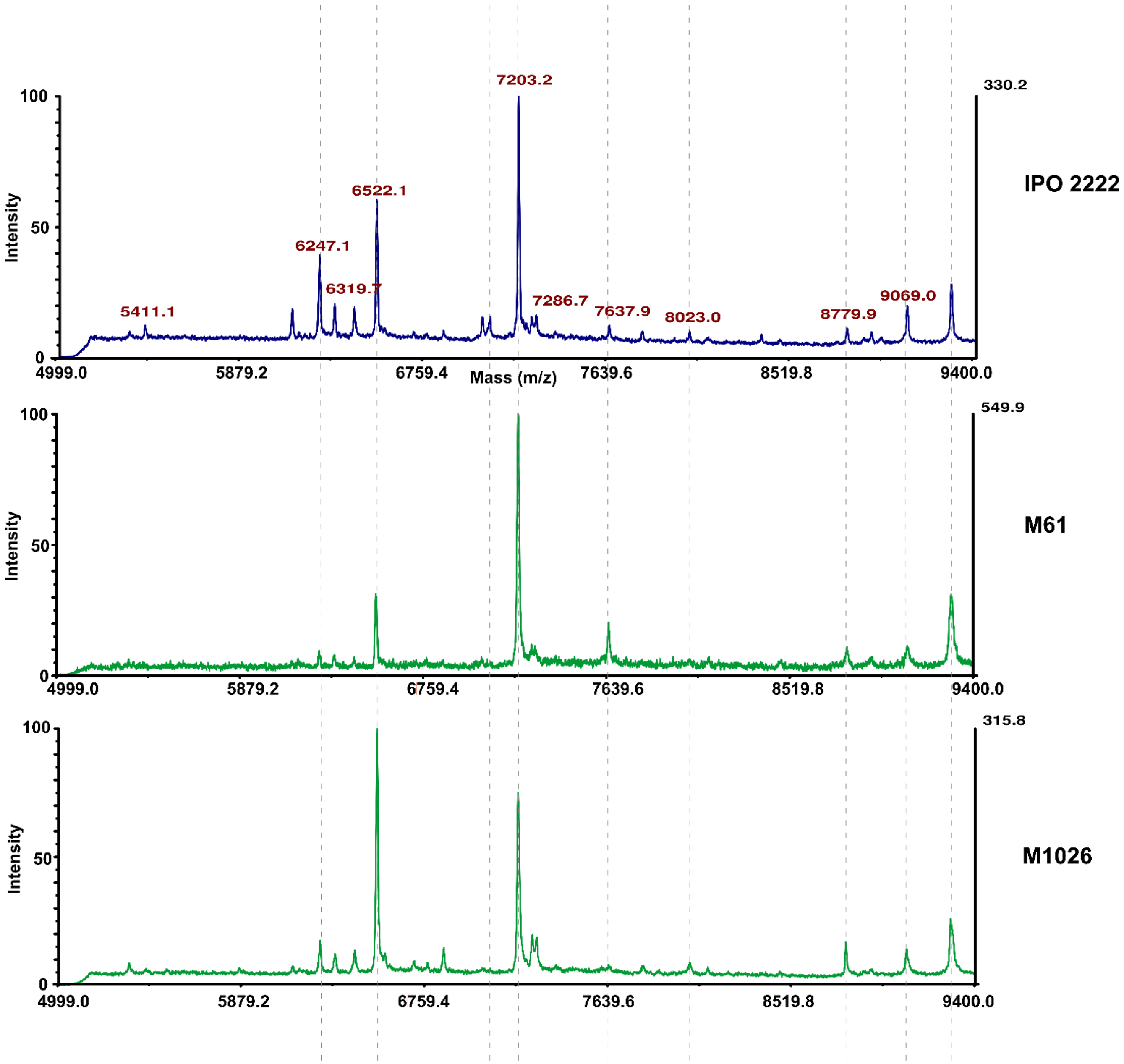
7203.2
330.2
100
50
0
Intensity
IPO 2222
6522.1
6247.1
6319.7
5411.1
7286.7
7637.9
8023.0
8779.9
9069.0
4999.0
5879.2
6759.4
Mass (m/z)
7639.6
8519.8
9400.0
549.9
M61
315.8
M1026

C)

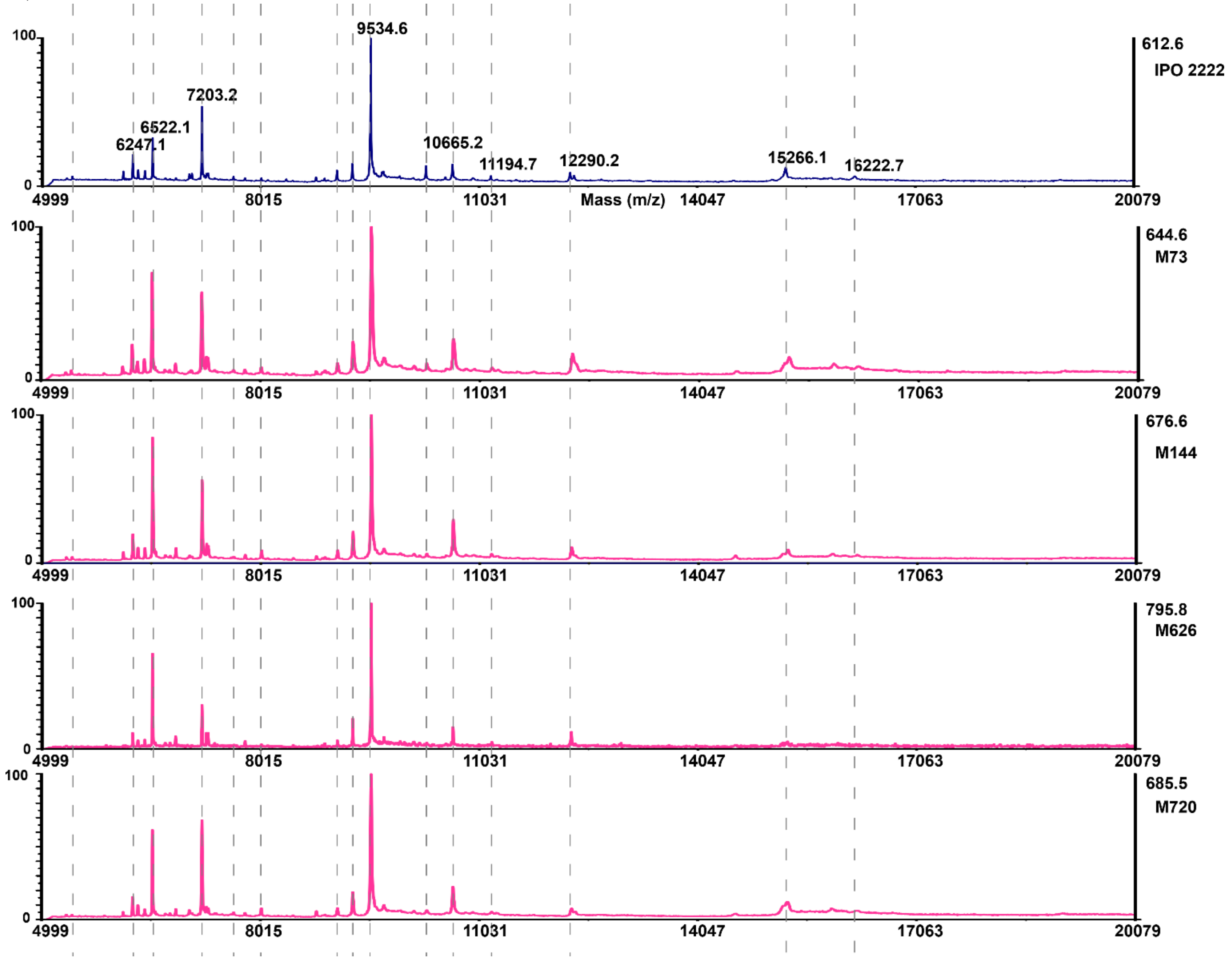

100
0
9534.6
7203.2
6522.1
6247.1
10665.2
11194.7
12290.2
15266.1
16222.7
4999
8015
11031
Mass (m/z)
14047
17063
20079
612.6
IPO 2222
644.6
M73
676.6
M144
795.8
M626
685.5
M720

D)

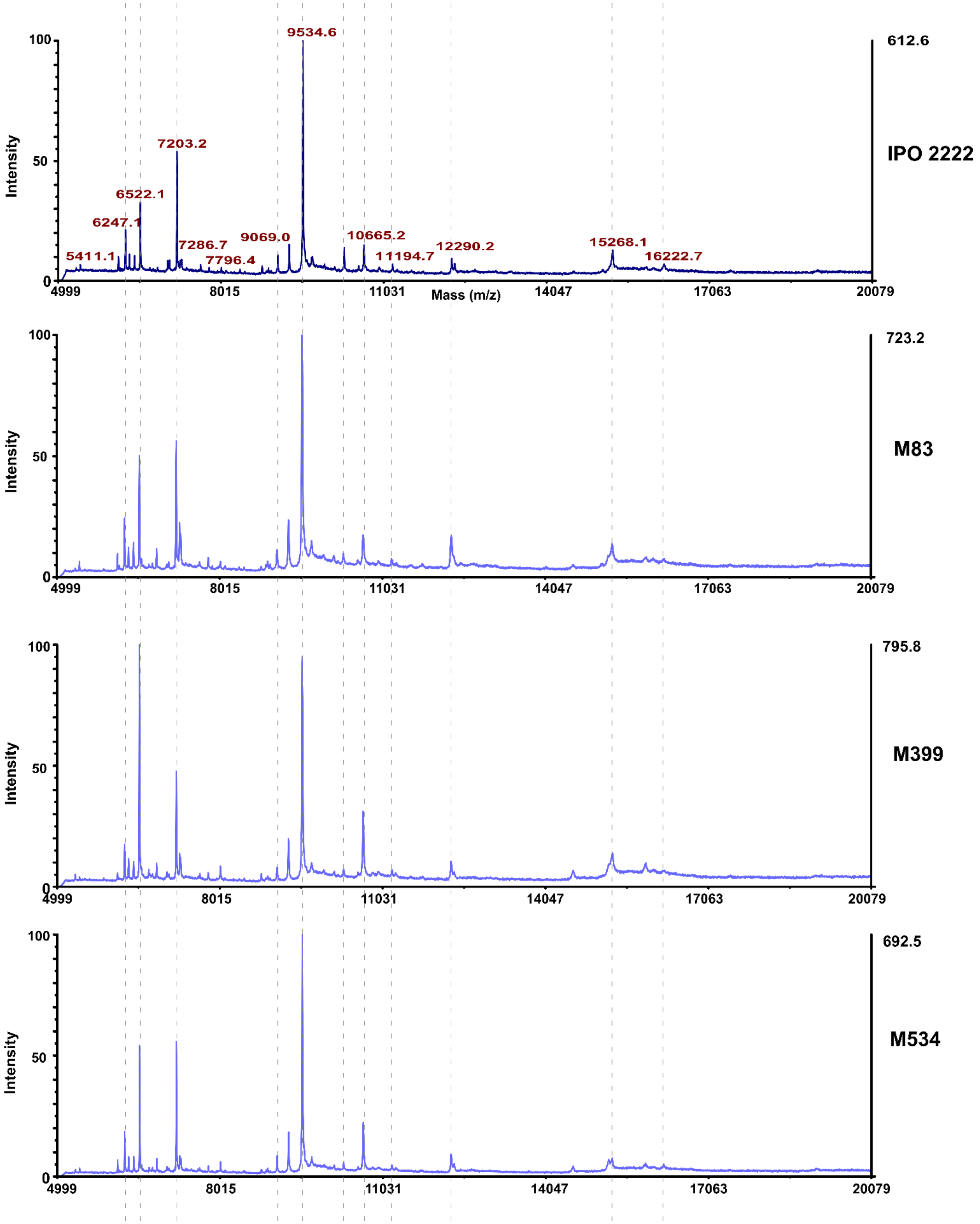

9534.6
7203.2
6522.1
6247.1
5411.1
7286.7
7796.4
9069.0
10665.2
11194.7
12290.2
15268.1
16222.7
Intensity
Mass (m/z)
612.6
IPO 2222
723.2
M83
795.8
M399
692.5
M534
4999
8015
11031
14047
17063
20079

E)

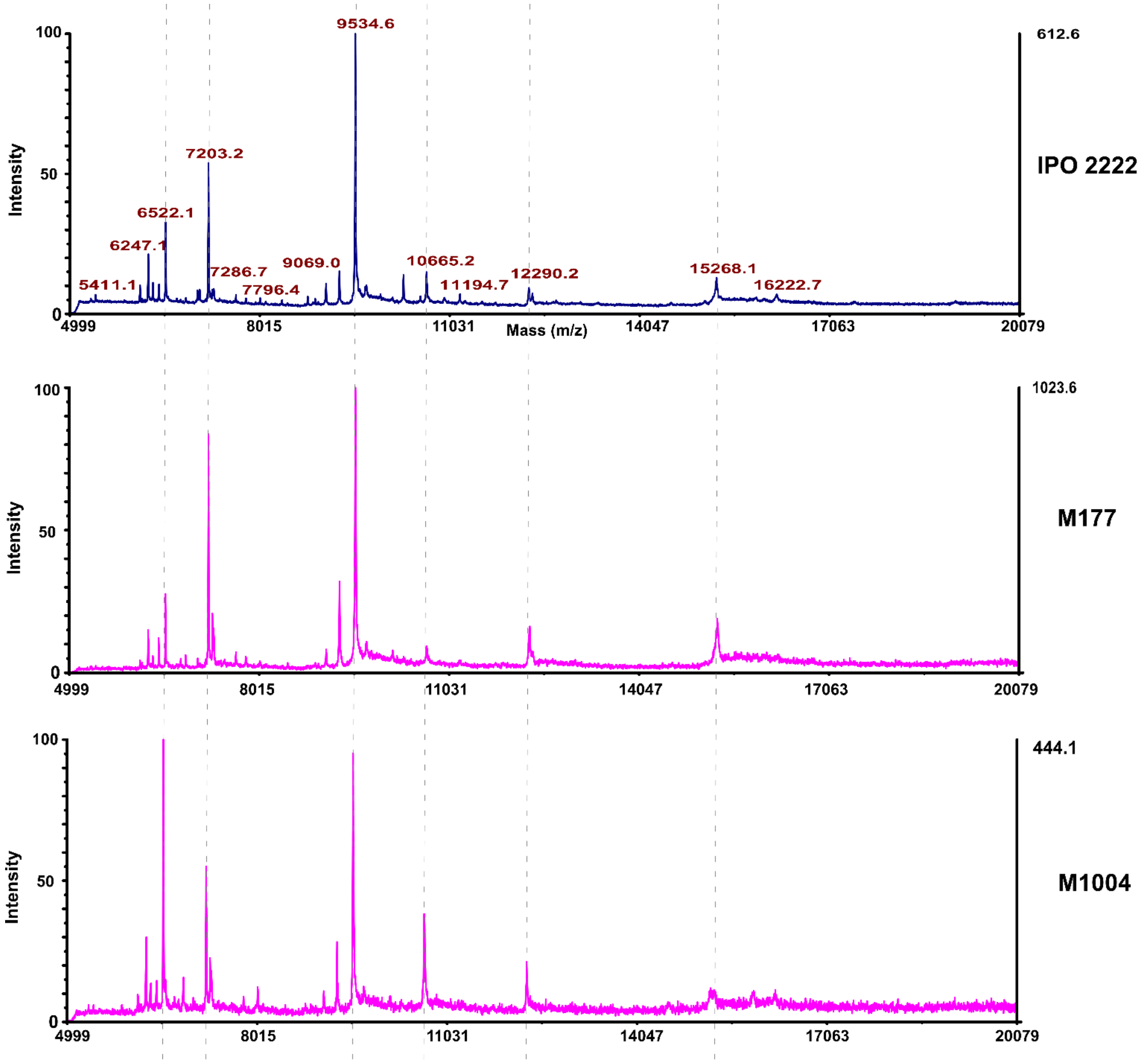

9534.6
612.6
100
50
0
Intensity
IPO 2222
7203.2
6522.1
6247.1
5411.1
7286.7
7796.4
9069.0
10665.2
11194.7
12290.2
15268.1
16222.7
4999
8015
11031
Mass (m/z)
14047
17063
20079
1023.6
M177
444.1
M1004
